# Sphingomonas longa sp. nov., Sphingomonas mollis sp. nov. and Sphingomonas aurea sp. nov.: three novel Sphingomonas species isolated from soil

**DOI:** 10.1099/ijsem.0.006572

**Published:** 2024-12-18

**Authors:** Eo Jin Kim, Soo Hyun Maeng, Myung Kyum Kim

**Affiliations:** 1 Department of Bio & Environmental Technology, College of Natural Science, Seoul Women’s University, Seoul 01797, Republic of Korea

**Keywords:** 16S rRNA gene sequencing, *Sphingomonadaceae*, *Sphingomonas*, taxonomy, whole-genome sequencing

## Abstract

Three Gram-negative, aerobic and non-motile bacterial strains, BT552^T^, BT553^T^ and KR1UV-12^T^, were isolated from soil samples in Gwangju-si and Gangneung-si, the Republic of Korea. Phylogenetic analysis based on 16S rRNA gene sequence showed that strains BT552^T^, BT553^T^ and KR1UV-12^T^ clustered to a distinct clade within the family *Sphingomonadaceae* (order *Sphingomonadales*, class *Alphaproteobacteria*). The strains exhibited the highest genetic similarity with representatives of the genus *Sphingomonas*; moreover, strains BT552^T^ and BT553^T^ tightly clustered with *Sphingomonas melonis* DAPP-PG 224^T^ (98.2 and 98.1 %) and *Sphingomonas aquatilis* JSS-7^T^ (98.1 and 98.0 %), while strain KR1UV-12^T^ clustered with *S. melonis* DAPP-PG 224^T^ (97.9%) and *Sphingomonas rubra* BH3^T^ (97.8%), respectively. The major cellular fatty acids of all three strains were summed feature 8 (C_18:1_ ω7c/C_18:1_ ω6c), comprising 44.7, 46.4 and 48.5%. Additionally, their respiratory quinone is Q-10, and polar lipids were diphosphatidylglycerol, phosphatidylglycerol, phosphatidylethanolamine, phospholipids, sphingolipid and phosphatidylcholine. They all grow well at an optimum temperature of 25 °C, at pH 7. The draft genomes of strains BT552^T^, BT553^T^ and KR1UV-12^T^ measures 4 035 561 bp, 3 941 714 bp and 3 418 792 bp, respectively, comprising 3 804 3648 and 3236 coding sequences and 50, 48 and 45 RNA genes. The average nucleotide identity analysis and digital DNA–DNA hybridization values between BT552^T^, BT553^T^ and KR1UV-12^T^ and closely related *Sphingomonas* species range from 72.7 to 80.2% and 19.4 to 24.3%, respectively. Based on phenotypic, genotypic and chemotaxonomic data, these three strains BT552^T^, BT553^T^ and KR1UV-12^T^ represent three novel bacterial species within the genus *Sphingomonas* for which the names *Sphingomonas longa* sp. nov. (type strain BT552^T^= KCTC 82094^T^ =NBRC 114993^T^), *Sphingomonas mollis* sp. nov. (type strain BT553^T^ =KCTC 82095^T^ =NBRC 114994^T^) and *Sphingomonas aurea* sp. nov. (type strain KR1UV-12^T^ = KCTC 92959^T^ = TBRC 18506^T^) are proposed.

## Introduction

The genus *Sphingomonas* is a recognized taxonomic group within the *Alphaproteobacteria* class, specifically belonging to *Sphingomonadaceae* family. The taxonomic classification of *Sphingomonas* was first defined by Yabuuchi et al. (1990), with *Sphingomonas paucimobilis* as the designated type species. By June 2024, the genus included 163 validly published species as per List of Prokaryotic names with Standing in Nomenclature (https://lpsn.dsmz.de/genus/sphingomonas) and have been isolated from diverse environments such as soil, water, plants and fruits [1,7]. The genus *Sphingomonas* is Gram-negative, aerobic and rod-shaped and forms yellow or orange colonies [8]. The chemotaxonomic characteristics of the *Sphingomonas* genus include sphingoglycolipid, phosphatidyl dimethylethanolamine, phosphatidylglycerol, phosphatidylcholine and phosphatidylethanolamine as the predominant polar lipids. The predominant respiratory quinone in the genus *Sphingomonas* is Q-10. The major cellular fatty acids are summed feature 3 (C_16:1_ ω7c/C_16:1_ ω6c), summed feature 8 (C_18:1_ ω7c/C_18:1_ ω6c) and C_14:0_ 2OH [9]. Most species of the genus *Sphingomonas* take sym-homospermidine as the major polyamine [10].

*Sphingomonas* exhibit remarkable capabilities that make them invaluable in various environmental and agricultural contexts. Their proficiency in degrading organic pollutants is particularly noteworthy, regarding their contribution to eliminating contaminated soils, water bodies and other environments. Moreover, in its involvement in processes like nitrogen fixation and denitrification, *Sphingomonas* species help ensure the availability of essential nutrients for plant growth, thereby contributing to higher agricultural productivity. Their biocontrol and stress tolerance abilities further highlight their importance in sustaining plant health and stability in the soil environment [8,11]. The numerous functional characteristics of *Sphingomonas* emphasize its potential as a significant participant in studies of the environment and biotechnological applications. This ability helps restore ecosystems and mitigates potential harm to human health and other organisms in the affected areas.

Additionally, the biocontrol mechanisms exhibited by *Sphingomonas* against pathogens and their tolerance to environmental stressors further enhance their utility in agriculture. By protecting plants from diseases and environmental stresses, these bacteria maintain crop health and stability, ultimately improving agricultural yields and resilience. The multifaceted functional characteristics of *Sphingomonas* make them essential subjects of scientific research and potential candidates for various biotechnological applications. Whether in environmental remediation, agriculture or beyond, their versatility and efficacy make them valuable assets in addressing contemporary challenges related to pollution, food security and sustainability [8,9, 11].

This work focuses on genomic analysis, taxonomic categorization and characterization of the recently obtained strains BT552^T^, BT553^T^ and KR1UV-12^T^, representing distinctive *Sphingomonas* feature*s*. Our research, especially the genomic insights, offers a deeper understanding of how these strains fulfil the key functional roles of *Sphingomonas* in soil environments. By analysing their genetic makeup, we clarify their capabilities in biodegradation, nutrient cycling and plant interaction, highlighting the importance of these strains in microbial taxonomy and biotechnological applications.

## Methods

### Organism and culture conditions

Strains BT552^T^ and BT553^T^ were isolated from soil in Gwangju-si (35° 9′ 34.363″ N and 126° 51′ 9.364″ E), whereas strain KR1UV-12^T^ was isolated from soil in Gangneung-si (37° 45′ 6.84″ N and 128° 52′ 41.7″ E), the Republic of Korea. One gram of soil was suspended in 10 ml of sterile normal saline, incubated at 37 °C for 1 h and then serially diluted. A 100 µl aliquot of the diluent was spread onto Reasoner’s 2A (R2A) (BD Difco) agar plates and incubated at 25 °C. After 3 days of incubation, several colonies were examined and selected for further purification. Among the isolated strains, BT552^T^, BT553^T^ and KR1UV-12^T^ were sub-cultured routinely and preserved at −80 °C in R2A broth containing 20% (v/v) glycerol. The closely related strains were obtained from the Korean Agricultural Culture Collection, Republic of Korea.

### Phylogenetic analysis and genome sequencing

The genomic DNA of strains BT552^T^, BT553^T^ and KR1UV-12^T^ was extracted using a Qiagen DNA extraction kit (Qiagen, Germany), following the manufacturer’s instructions. The 16S rRNA gene was amplified via standard PCR with the bacterial primer pairs 27F and 1492R described by Weisburg et al. [12]. The purified PCR products were sequenced by Bionics (Republic of Korea). The taxonomic classification of the strains was determined by comparing their 16S rRNA gene sequences on the EzBioCloud database (https://www.ezbiocloud.net). Sequences of phylogenetically related taxa were retrieved from the EzBioCloud database. Additionally, NCBI blast (basic local alignment search tool) (https://blast.ncbi.nlm.nih.gov/Blast.cgi) was used to obtain updated information unavailable on the EzBioCloud. The 16S rRNA gene sequences of BT552^T^, BT553^T^ and KR1UV-12^T^ and the closely related strains were aligned using the EzEditor2 program. Phylogenetic trees were constructed using mega 11 software [13], employing three algorithms: neighbour joining (NJ) by Saitou and Nei [14], maximum likelihood (ML) by Felsenstein [15] and maximum parsimony (MP) by Fitch [16]. Bootstrap analysis with 1000 replicates, as introduced by Felsenstein [17], was performed to assess the reliability of the trees.

Genomic DNA from strains BT552^T^, BT553^T^ and KR1UV-12^T^ was extracted using the Solgent genomic DNA extraction kit (Solgent, South Korea). After extraction, the concentration of DNA was measured, and Illumina’s Nextera DNA Flex Library Prep Kit was used to construct and generate DNA libraries. Whole-genome sequencing was conducted on the iSeq 100 platform, producing 150 bp paired-end reads. The sequences were assembled using SPAdes (v.3.13.0), developed by the Algorithmic Biology Lab at the St. Petersburg Academic University of the Russian Academy of Sciences [18].

The genome sequences of strains BT552^T^, BT553^T^ and KR1UV-12^T^ have been submitted to the GenBank database (www.ncbi.nlm.nih.gov/) and annotated using the Prokaryotic Genome Annotation Pipeline provided by the National Center for Biotechnology Information (NCBI). The genomic distance between the strains was calculated by the average nucleotide identity (ANI) using the EzBioCloud web tool (https://www.ezbiocloud.net/tools/ani) [19]. The digital DNA–DNA hybridization (dDDH) values were calculated using the Genome-to-Genome Distance Calculator, explicitly utilizing formula 2 to enhance precision in the calculation [20].

For the taxonomic classification of the bacterial genomes of novel bacteria, the Genome Taxonomy Database Toolkit (GTDB-Tk) (v.2.3.0) was used to generate a GTDB tree and approximate the taxonomy of the novel bacteria (https://github.com/Ecogenomics/GTDBTk). This method involved a concatenated multiple-sequence alignment of 120 marker genes. The resulting tree was visualized using iTOL version 4 (https://itol.embl.de).

Whole-genome assemblies for novel strains and their closely related type strains, as identified in the GTDB tree, were used to reconstruct a whole-genome-based phylogenetic tree using the up-to-date bacterial core gene (UBCG) set pipeline. This approach, detailed by Na et al. [21], leverages a concatenated sequence dataset of 92 single-copy bacterial core genes (www.ezbiocloud.net/tools/ubcg) for phylogenetic reconstruction. Furthermore, functional genes were analysed and categorized to evaluate these strains’ metabolic characteristics and ecological roles using the Rapid Annotation using Subsystem Technology (RAST) server [22,23].

### Morphological, physiological and biochemical analysis

The cell morphology was examined using transmission electron microscopy (JEOL, JEM1010) after incubating for 3 days on R2A agar plates at 25 °C. The Gram staining of strains BT552^T^, BT553^T^ and KR1UV-12^T^ was performed using a standard Gram reaction kit by bioMérieux. Growth conditions were tested at various temperatures: 4, 10, 15, 25, 30, 35 and 40 °C. The pH tolerance range was determined using nutrient agar (NA), with pH values from 5.0 to 9.0 in 0.5 unit increments, all maintained at 25 °C. pH-dependent growth was assessed with two different buffers at a final concentration of 100 mM: acetate buffer for pH 5.0–6.5 and phosphate buffer for pH 7.0–9.0. NaCl tolerance concentrations ranged from 1 to 10% (w/v at 1% intervals). Oxidase activity was assessed using a 1 % (w/v) tetramethyl-*p*-phenylenediamine solution [24], and catalase activity was evaluated by observing bubble production after applying a 3% (v/v) hydrogen peroxide solution [25]. Growth on various culture media was observed on R2A agar, NA (BD Difco), tryptic soy agar (TSA) (BD Difco), MacConkey agar (BD Difco) and lysogeny broth (LB) (BD Difco). Carbon source utilization and fermentation were determined using the API 20NE test kit, and enzymatic activities were assessed using the API ZYM test kit (bioMérieux) according to the manufacturer’s guidelines.

### Chemotaxonomic characteristics

Polar lipids from strains BT552^T^, BT553^T^ and KR1UV-12^T^ were extracted following the method described by Minnikin et al. [26] and analysed using two-dimensional TLC. The separated polar lipids were identified with a series of reagents, using a mixture of chloroform, methanol and water in ratios of 9:10:3 v/v/v for the first dimension and 5:10:4 v/v/v for the second (Komagata and Suzuki 1987) [27]. For identifying total lipids, glycolipids, sphingolipids, glycosphingolipids, phosphatidylcholine, amino groups and phosphorus-containing lipids, the TLC plate was treated with ethanolic molybdatophosphoric acid (Merck), α-naphthol in sulfuric acid reagent (Wako), Dragendorff’s reagent (Merck), a ninhydrin spray solution (Merck) and molybdenum blue reagent (Sigma). Quinones of strains BT552^T^, BT553^T^ and KR1UV-12^T^ were extracted using Sep-Pak Vac cartridges (Waters) and analysed by HPLC [28].

For cellular fatty acid and polyamine analysis, strains BT552^T^, BT553^T^ and KR1UV-12^T^ were cultured on NA for 3 days at 25 °C. Cellular fatty acids were purified via saponification, methylation and extraction procedures, according to Sasser [29]. The resulting fatty acid methyl esters were identified using the Sherlock Microbial Identification System V6.01 (MIS, database TSBA6; MIDI Inc., Newark, DE). Polyamines were extracted and analysed according to the methods described by Busse and Auling [30] and Busse et al. [31].

### Results and discussion

### Morphological, physiological and biochemical analyses

**Fig. 1.**
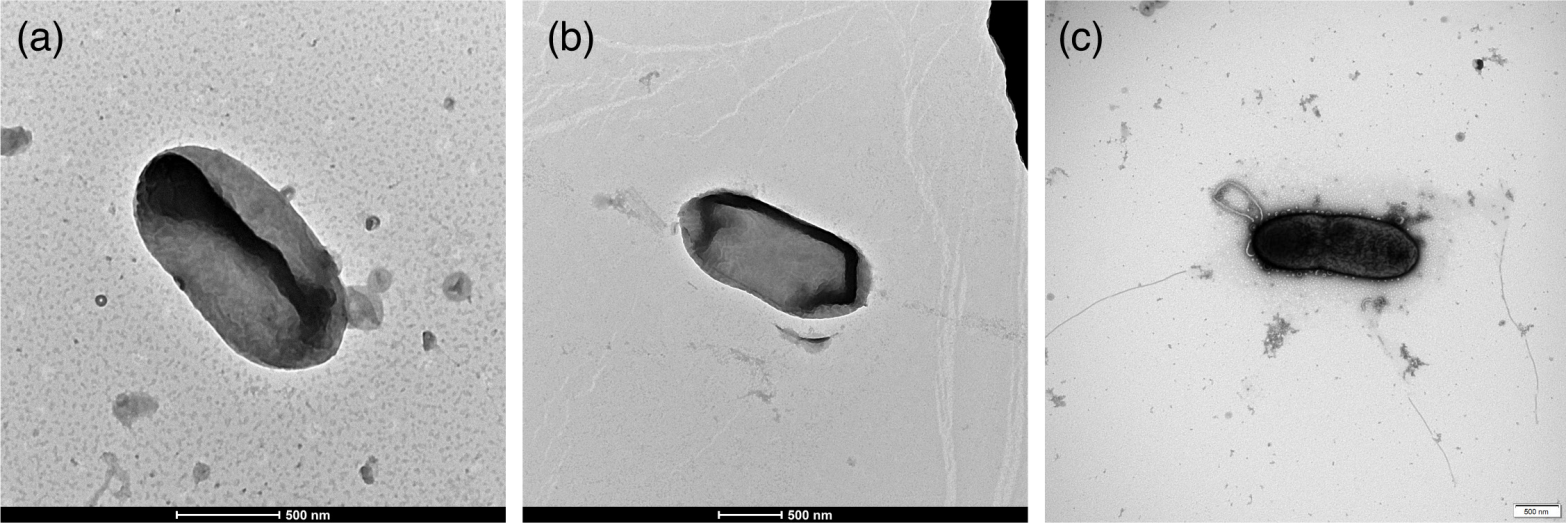
Transmission electron micrographs of strains BT552^T^ (**a**), BT553^T^ (**b**) and KR1UV-12^T^ (**c**).

The strains BT552^T^, BT553^T^ and KR1UV-12^T^ were Gram-negative, non-motile and rod-shaped (Fig. 1). The colonies of strains BT552^T^ and BT553^T^ exhibited a yellow colour, convex and round after 72 h of incubation on NA at 30 °C. Similarly, strain KR1UV-12^T^ exhibited an orange-yellow colour with rounded, slightly convex-shaped colonies after 72 h of incubation on NA at 30 °C. BT552^T^ and KR1UV-12^T^ strains grew at 20–30 °C (optimum at 25 °C) without NaCl. However, strain BT553^T^ grew within a temperature range of 10–30 °C (optimum at 25 °C) in the absence of NaCl. Although BT552^T^ and BT553^T^ have similar colony morphology, BT552^T^ shows positive reactions for gelatin hydrolysis and α-chymotrypsin activity, while BT553^T^ shows negative reactions, indicating differences in biochemical characteristics between these strains. Table 1 lists the distinct physical characteristics that distinguish strains BT552^T^, BT553^T^ and KR1UV-12^T^ and their closest neighbours in the *Sphingomonas* genus.

**Table 1.** Differential characteristics of strains BT552^T^, BT553^T^ and KR1UV-12^T^ and closely related species Taxa: 1, strain BT552^T^; 2, strain BT553^T^; 3, strain KR1UV-12^T^; 4, *S. melonis* DAPP-PG 224^T^; 5, *S. aquatilis* JSS-7^T^; 6, *S. rubr*a BH3^T^; 7, *S. liriopis* RP10^T^; 8, *S. cynarae* SPC-1^T^; 9, *S. metalli* 9O-1^T^. Data of strains BT552^T^ and BT553^T^ and reference strains were obtained in this study unless indicated otherwise. +, Positive; −, negative; w, weak positive; nd, no data. All strains had positive results for aesculin hydrolysis. All strains were negative for indole production and acid production from glucose and urease.

Characteristic	1	2	3	4	5	6	7	8	9
Characteristic morphology	Convex, smooth	Convex, smooth	Slightly convex, glistening	Domed, smooth	Low-convex, smooth, opaque	Elevated, circular	Convex, smooth	Convex, smooth	Opaque, convex
Colony colour	Yellow	Yellow	Orange yellow	Deep yellow	Yellow	Red	Light yellow	Bright orange	Orange
Nitrate reduction to NO_2_	−	−	−	−	−	nd	+	−	−
Nitrate reduction to N_2_	−	−	−	nd	−	nd	+	−	−
**Enzyme activity**									
Arginine dihydrolase	−	−	−	−	−	−	+	−	−
Gelatin hydrolysis	+	−	+	−	−	−	−	+	+
*N*-Acetyl-*β*-glucosaminidase	w	+	+	+	+	−	−	nd	nd
Trypsin	w	−	w	+	+	−	+	nd	nd
*α*-Chymotrypsin	w	−	+	−	−	+	−	nd	nd
*β*-Galactosidase (ONPG)	+	+	+	+	+	−	−	nd	nd
**Assimilation**									
Adipate	−	−	−	−	nd	−	−	−	−
Caprate	−	−	−	−	+	−	−	−	−
Citrate	−	−	−	−	−	nd	−	−	−
d-Glucose	−	−	−	+	+	w	+	+	+
d-Maltose	−	−	−	+	+	nd	+	+	+
d-Mannitol	−	−	−	−	nd	nd	−	+	−
d-Mannose	−	−	−	+	−	nd	−	+	+
Gluconate	−	−	−	−	+	−	−	+	+
l-Arabinose	−	−	−	+	+	nd	−	+	+
l-Malate	−	−	−	nd	−	nd	+	−	+
*N*-Acetyl-d-glucosamine	−	−	−	+	+	−	−	−	−
Phenyl acetate	−	−	−	+	−	−	−	−	−
G+C content	66.8	65.9	68.3	67.0*	67.1	68.8†	68.2	66.6‡	68.6§

*Data from Buonaurio et al. [1]; †data from Huo et al. [2]; ‡data from Talà et al. [32]; §data from Feng et al. [10,33].

### Phylogenetic analysis and genome sequencing

**Fig. 2.**
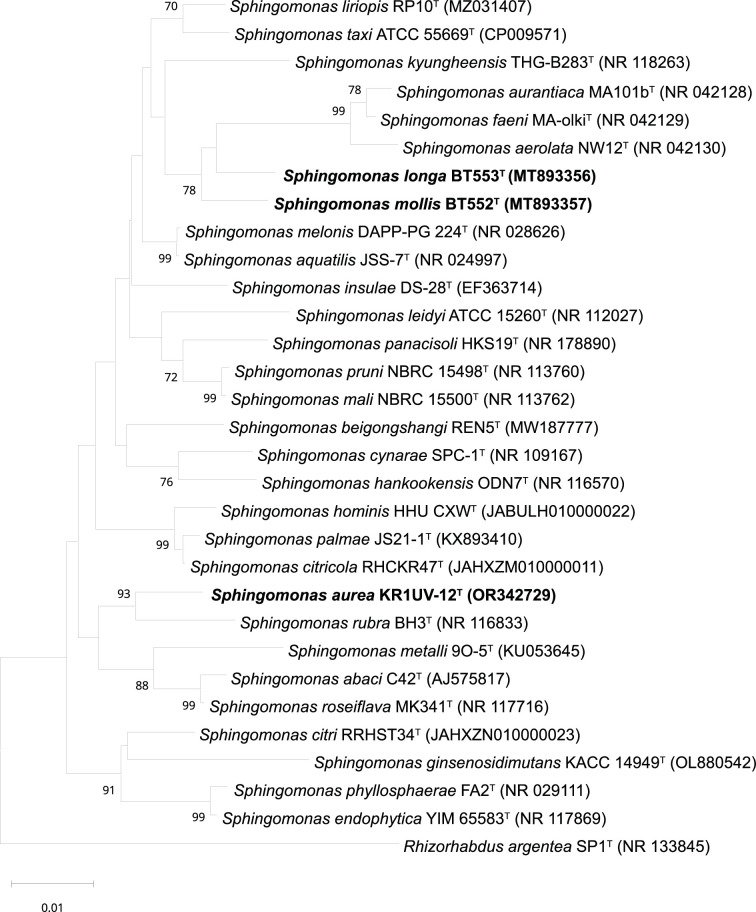
NJ phylogenetic tree based on 16S rRNA gene sequences showing the position of strains BT552^T^, BT553^T^ and KR1UV-12^T^ and other relative species of the genus *Sphingomonas*. Numbers at nodes are bootstrap percentages (> 70%) based on the NJ algorithms. *Rhizorhabdus argentea* SP1^T^ was used as an outgroup. [34] Bar, 0.01 substitutions per nucleotide position.

In the context of phylogenetic classification, the comparative study of strains BT552^T^, BT553^T^ and KR1UV-12^T^ utilizing 16S rRNA gene sequencing and comprehensive genome analysis has produced significant results. The analysis classified these strains as members of the *Sphingomonadaceae* family. The 16S rRNA gene sequence comparisons indicate that these strains belong to the genus *Sphingomonas*. This alignment is further corroborated by genome-based phylogenetic analysis, providing a cohesive view of their taxonomic placement. These findings affirm the placement of strains BT552^T^, BT553^T^ and KR1UV-12^T^ within the genus *Sphingomonas*, contributing to the taxonomic clarity of the *Sphingomonadaceae* family. The 16S rRNA gene sequence of strain BT552^T^ showed high similarity to *Sphingomonas melonis* DAPP-PG 224^T^ (98.2%), *Sphingomonas aquatilis* JSS-7^T^ (98.1%), *Sphingomonas kyungheensis* THG-B283^T^ (97.7%) and *Sphingomonas liriopis* RP10^T^ (97.6%). Strain BT553^T^ showed the highest similarity to *S. melonis* DAPP-PG 224^T^ (98.1%), *S. aquatilis* JSS-7^T^ (98.0%), *S. liriopis* RP10^T^ (97.8%) and *Sphingomonas taxi* ATCC 55669^T^ (97.6%). Strain KR1UV-12^T^ showed the highest similarity to *S. melonis* DAPP-PG 224^T^ (97.9%), *Sphingomonas rubra* BH3^T^ (97.8%), *S. liriopis* RP10^T^ (97.8%) and *S. aquatilis* JSS-7^T^ (97.8%). The strains used are listed in Table S1 (available in the online Supplementary Material). Phylogenetic trees were constructed using the NJ method (Fig. 2), ML method (Fig. S1) and MP method (Fig. S2).

Genomic sequencing revealed that the genome of strain BT552^T^ spans 4 035 561 base pairs, with a sequencing coverage of 54.1×. This genome comprises 3804 coding sequences (CDSs). The calculated DNA G+C content is 66.8%, which falls within the typical range for the genus *Sphingomonas* (58.7 –71.0 %), as referenced in the NCBI genome database (https://www.ncbi.nlm.nih.gov/genome/browse). In a parallel analysis, the genome of strain BT553^T^ was determined to encompass 3 941 714 base pairs, with a genome coverage of 55.3×. This genome is characterized by 3648 CDSs and a DNA G+C content of 65.9%, aligning with the known range for *Sphingomonas* species. Lastly, the genome of strain KR1UV-12^T^ was determined to encompass 3 418 792 base pairs, with a genome coverage of 59.3×. This genome is characterized by 3236 CDSs and a DNA G+C content of 68.3%, also within the known range for *Sphingomonas* species. The genome sequences of strains BT552^T^, BT553^T^ and KR1UV-12^T^ have been deposited in the DDBJ/EMBL/GenBank databases under the accession numbers JAFEMC000000000, JAELXS000000000 and JAUUDS000000000, respectively. Genome sequences of closely related species were sourced from NCBI for comparative purposes. The details of these genomes and their assembly statistics are comprehensively listed in Tables S2 and S3.

The genomic comparisons between strain BT552^T^ and other members of the *Sphingomonas* genus yielded ANI values ranging from 72.8 to 77.6% and dDDH values between 19.9 and 22.1%. For strain BT553^T^, the ANI values with other *Sphingomonas* species were between 72.8 and 79.9 %, with dDDH values ranging from 19.4 to 23.7%. For strain KR1UV-12^T^, the ANI values with other *Sphingomonas* species were between 73.2 and 80.2%, with dDDH values ranging from 20.1 to 24.3%. As part of our internal analysis, when comparing distance, ANI and dDDH among the newly identified strains BT552^T^, BT553^T^ and KR1UV-12^T^, the distance between BT552^T^ and BT553^T^ was calculated to be 0.2017, with an ANI value of 77.3% and a dDDH value of 21.7%. Similarly, the distance between BT552^T^ and KR1UV-12^T^ was 0.1978, with an ANI value of 77.8% and a dDDH value of 22.2%. The distance between BT553^T^ and KR1UV-12^T^ was 0.2010, with an ANI value of 77.9% and a dDDH value of 21.8%. Notably, BT552^T^, BT553^T^ and KR1UV-12^T^ displayed ANI values significantly below the conventional species delineation threshold of 95–96%. Similarly, the dDDH values for these strains were well below the established criterion for species differentiation in prokaryotes, which is ≥70% in *in silico* DDH. The comprehensive genome comparisons of strains BT552^T^, BT553^T^ and KR1UV-12^T^ are detailed in Tables S4, S5 and S6. These findings are critical in establishing the taxonomic uniqueness of strains BT552^T^, BT553^T^ and KR1UV-12^T^ within the genus *Sphingomonas*.

The results of phylogenetic analysis based on the 16S rRNA gene and whole-genome sequences revealed relationships for these strains. When analysing the phylogenetic relationships based on the 16S rRNA gene, strains BT552^T^ and BT553^T^ showed the closest phylogenetic affinity to *S. melonis* and *S. aquatilis*. In contrast, strain KR1UV-12^T^ showed a closer relationship to *S. rubra* and *Sphingomonas criticola* (Figs 2, S1 and S2). Further, in the reconstructed phylogenetic analysis using the genome-based UBCG tool, strains BT552^T^ and BT553^T^ were closely aligned with *Sphingomonas cynarae* and *S. rubra*. However, the phylogenetic positioning of BT552^T^ and BT553^T^ is sufficiently unique to support their classification as new species within the genus *Sphingomonas*. In contrast, the UBCG phylogenetic tree places strain KR1UV-12^T^ closest to *Sphingomonas metalli* (Fig. S3). Despite the variations in the closest species determined by genome-based phylogenetic analyses, KR1UV-12^T^ showed enough phylogenetic distinction to be proposed as a new species of the *Sphingomonas genus*. These genomic results support the proposal to classify strains BT552^T^, BT553^T^ and KR1UV-12^T^ as novel species in the *Sphingomonas* genus.

The RAST server comprehensively annotated the genomes of strains BT552^T^, BT553^T^ and KR1UV-12^T^. For strain BT552^T^, the RAST analysis identified a total of 275 subsystems (Fig. S4). Similarly, the genome of strain BT553^T^ contained 291 subsystems (Fig. S5), indicating a broad repertoire of biological functions. For strain KR1UV-12^T^, the RAST analysis identified a total of 268 subsystems (Fig. S6), suggesting a complex genomic architecture with diverse functional capabilities. These annotations provide insights into the novel strains’ metabolic diversity. The RAST annotation details are accessible at https://rast.nmpdr.org/. Using the blastp algorithm, we compared strains BT552^T^, BT553^T^ and KR1UV-12^T^ to related *Sphingomonas* species, focusing on genes for auxin biosynthesis and ammonia assimilation. Strain BT552^T^ showed 71.4–93.1 % similarity in auxin biosynthesis genes (Table S7), and BT553^T^ had 73.0–93.6% similarity (Table S8). KR1UV-12^T^ exhibited 72.9–93.2% similarity, suggesting conserved pathways with unique strain-specific features (Table S9). For ammonia assimilation genes, BT552^T^ showed 0–97.3% similarity (Table S10), BT553^T^ ranged from 0 to 99.1% (Table S11) and KR1UV-12^T^ had 0–99.1% similarity (Table S12), indicating diverse nitrogen metabolism. These sequence differences and phylogenetic analyses support the classification of strains BT552^T^, BT553^T^ and KR1UV-12^T^ as novel species within the *Sphingomonas* genus.

### Chemotaxonomic characterization

The chemotaxonomic analyses were conducted to determine the cellular fatty acid compositions of strains BT552^T^, BT553^T^ and KR1UV-12^T^, alongside comparisons with their nearest phylogenetic neighbours (Table 2). For BT552^T^, the predominant fatty acids were summed feature 8 (comprising C_18:1_ ω7c and C_18:1_ ω6c), summed feature 3 (comprising C_16:1_ ω7c and C_16:1_ ω6c) and C_14:0_ 2OH, constituting 77.84% of the fatty acid profile. This strain was distinguishable from closely related types by an increased presence of summed feature 3. Similarly, BT553^T^ and KR1UV-12^T^ were characterized by substantial amounts of summed feature 8, summed feature 3 and a significant proportion of C_14:0_ 2OH. The polar lipid assays revealed that all three strains possess lipids, diphosphatidylglycerol (DPG), phosphatidylglycerol (PG), phosphatidylethanolamine (PE), phosphatidylcholine (PC), phospholipids (PL) and sphingolipid (SL) with BT552^T^ exhibiting additional complex lipids, including aminolipid and aminophospholipid (Figs S7, S8 and S9). The presence of sphingolipids in all strains is a chemotaxonomic marker consistent with the *Sphingomonas* genus. The respiratory quinone profile, dominated by ubiquinone Q-10 in all three isolates, corroborates their placement within the genus. The major polyamine of BT552^T^, BT553^T^ and KR1UV-12^T^ are homospermidine. BT552^T^ and KR1UV-12^T^ strains contain putrescine as their minor polyamine, constituting 6.48 and 14.66% of the total polyamines. In contrast, BT553^T^ differs by having spermidine as its minor polyamine, accounting for 1.93% (Fig. S10). These chemotaxonomic signatures and the phylogenetic data support the classification of strains BT552^T^, BT553^T^ and KR1UV-12^T^ as novel entities within the *Sphingomonas* genus.

**Table 2.** Cellular fatty acid profiles of strains BT552^T^, BT553^T^ and KR1UV-12^T^ and closely related species Taxa: 1, strain BT552^T^; 2, strain BT553^T^; 3, strain KR1UV-12^T^; 4, *S. melonis* DAPP-PG 224^T^; 5, *S. aquatilis* JSS-7^T^; 6, *S. rubra* BH3^T^; 7, *S. liriopis* RP10^T^; 8, *S. metalli* 9O-1^T^; 9, *S.cynarae* SPC-1^T^. Data of strains BT552^T^, BT553^T^ and KR1UV-12^T^ and reference strains were obtained in this study. tr, trace (< 1 %); nd, not detected.

Fatty acid	1	2	3	4	5	6	7	8	9
**Saturated**									
C_14:0_	tr	4.86	1.23	2	tr	tr	tr	nd	tr
C_16:0_	8.54	8.41	8.13	19.8	20.58	11.2	15.6	4.8	18.4
C_18:0_	tr	tr	tr	4.8	1.04	tr	3.4	nd	8.1
**Unsaturated**									
C_16:1_ ω5c	6.99	1.18	1.73	nd	tr	tr	tr	1.8	nd
C_17:1_ ω6c	tr	1.5	2.96	8.2	nd	2.2	1.8	nd	nd
C_17:1_ ω7c	nd	nd	nd	tr	nd	nd	1.0	nd	nd
C_18:1_ ω5c	tr	tr	1.07	nd	1.51	nd	nd	1.4	nd
C_18:1_ ω7c	nd	nd	nd	nd	nd	nd	nd	63.1	40.2
C_18:3_ ω6, 9, 12c	tr	nd	nd	nd	nd	nd	4.6	nd	nd
**Branched-chain fatty acid**									
C_18:1_ ω7c 11-methyl	4.49	3.15	3.24	5.9	nd	3.9	3.8	3.1	nd
**Hydroxy fatty acids**									
C_13:0_ 2OH	nd	nd	tr	1.6	nd	nd	1.9	nd	nd
C_14:0_ 2OH	12.5	11.69	14.95	6.3	10.86	20.6	9.7	11.3	5.4
C_15:0_ 2OH	tr	tr	0.43	nd	nd	tr	nd	nd	nd
C_18:1_ 2OH	tr	tr	tr	1.5	nd	nd	nd	nd	nd
**CYCLO**									
C_17:0_ cyclo	nd	nd	nd	nd	nd	nd	1.1	nd	2.0
C_19:0_ cyclo ω8c	nd	tr	nd	nd	nd	nd	1.2	nd	nd
**Summed feature**									
** 3**; C_16:1_ ω7c/C_16:1_ ω6c	17.67	19.77	16.21	5.5	4.61	18.9	nd	nd	nd
** 8**; C_18:1_ ω7c/C_18:1_ ω6c	44.69	46.38	47.48	35.6	59.89	39.2	32.8	nd	nd
** 9**; C_17:1_ iso ω9c/C_16:0_ 10-methyl	nd	tr	nd	tr	nd	nd	2.1	nd	nd

The results from the phylogenetic analysis distinctly indicated that strains BT552^T^, BT553^T^ and KR1UV-12^T^ represent new species within the genus *Sphingomonas*. Based on phenotypic, genotypic and biochemical characteristics, strains BT552^T^, BT553^T^ and KR1UV-12^T^ represent three novel species within the genus *Sphingomonas*. We propose the names *Sphingomonas longa*, *Sphingomonas mollis* and *Sphingomonas aurea* for these novel species.

## Description of *Sphingomonas longa* sp. nov.

*Sphingomonas longa* (lon’ga. L. fem. adj. *longa*, long, extended, referring to the length of cells of the type strain).

Cells are Gram-negative, non-motile and rod-shaped. Colonies grown on NA have a convex, circular and smooth appearance with a yellow colour after 72 h of incubation at 30 °C. The cells are ~0.6–1.5 µm in width and 1.5–5.8 µm in length. They grow on NA agar at 25–30 °C (optimum at 25 °C) and tolerate NaCl concentrations of 0–2.5 % (optimal at 0%). The strain grows on NA, TSA, LB and R2A agar but not on MAC agar. Both oxidase and catalase activities are positive. In the API 20NE test, positive reactions for *β*-glucosidase (aesculin hydrolysis), protease (gelatin hydrolysis) and *β*-galactosidase (PNPG). It tests negative for nitrate reduction, indole production and acid production from glucose, arginine dihydrolase, urease, d-glucose, l-arabinose, d-mannose, d-mannitol, *N*-acetyl-d-glucosamine, d-maltose, potassium gluconate, caprate, adipate, l-malate, citrate and phenylacetate. In the API ZYM test, strain BT552^T^ is positive for alkaline phosphatase, leucine arylamidase, valine arylamidase, acid phosphatase, naphtol-AS-BI-phosphohydrolase and *β*-galactosidase (ONPG). It exhibits weakly positive reactions for esterase (C4), esterase (C8), lipase (C14), cystine arylamidase, trypsin, *α*-chymotrypsin, *α*-galactosidase, *α*-glucosidase (starch hydrolysis), *β*-glucosidase and *N*-acetyl-*β*-glucosaminidase. It tests negative for *β*-glucuronidase, *α*-mannosidase and *α*-fucosidase.

The major respiratory quinone is Q-10. Dominant cellular fatty acids include summed feature 3 (C_16:1_ ω7c/C_16:1_ ω6c) and summed feature 8 (C_18:1_ ω7c/C_18:1_ ω6c). The major polyamine is homospermidine (93.5%), with putrescine (6.5%) as the minor polyamine. The major polar lipids in this strain include DPG, PG, PE, PC, PL and SL.

The type strain for *Sphingomonas longa*, BT552^T^ (=KCTC 82094^T^ = NBRC 114993^T^), was isolated from soil in the Republic of Korea. The genome sequence of strain BT552^T^ has been deposited in GenBank/DDBJ/EMBL under the accession number JAFEMC000000000. The GenBank accession number for the 16S rRNA gene sequence of strain BT552^T^ is MT893356.

## Description of *Sphingomonas mollis* sp. nov.

*Sphingomonas mollis* (mol’lis. L. fem. adj. *mollis*, soft).

Cells are Gram-negative, non-motile and rod-shaped. Colonies grown on NA have a convex, circular and smooth appearance with a yellow colour after 72 h of incubation at 30 °C. The cells are ~1.5 µm in width and 5.8 µm in length. They grow on NA at temperatures ranging from 10 to 30 °C (optimum at 25 °C) and tolerate NaCl concentrations of 0–2.5 % (optimal at 0%). The strain grows on NA, TSA, LB and R2A agar but not MAC agar. Oxidase and catalase activities are positive. In the API 20NE test, positive reactions for *β*-glucosidase (aesculin hydrolysis) and *β*-galactosidase (PNPG). It tests negative for nitrate reduction, indole production, acid production from glucose, arginine dihydrolase, urease, protease (gelatin hydrolysis), d-glucose, l-arabinose, d-mannose, d-mannitol, *N*-acetyl-d-glucosamine, d-maltose, potassium gluconate, caprate, adipate, l-malate, citrate and phenylacetate. In the API ZYM test, strain BT553^T^ is positive for alkaline phosphatase, leucine arylamidase, valine arylamidase, acid phosphatase, naphtol-AS-BI-phosphohydrolase, *β*-galactosidase (ONPG) and *N*-acetyl-*β*-glucosaminidase. It exhibits weakly positive reactions for esterase (C4), esterase (C8), cystine arylamidase, *α*-galactosidase, *β*-glucuronidase, *α*-glucosidase (starch hydrolysis), *β*-glucosidase and *α*-fucosidase. It tests negative for lipase (C14), trypsin, *α*-chymotrypsin and *α*-mannosidase.

The major respiratory quinone is Q-10. Dominant cellular fatty acids include summed feature 3 (C_16:1_ ω7c/C_16:1_ ω6c) and summed feature 8 (C_18:1_ ω7c/C_18:1_ ω6c). The major polyamine is homospermidine (98.07%), with spermidine (1.93%) as the minor polyamine. The major polar lipids in this strain include DPG, PG, PE, PC and SL.

The type strain for *Sphingomonas mollis*, BT553^T^ (=KCTC 82095^T^ = NBRC 114994^T^), was isolated from soil in the Republic of Korea. The genome sequence of strain BT553^T^ has been deposited in GenBank/DDBJ/EMBL under the accession number JAELXS000000000. The GenBank accession number for the 16S rRNA gene sequence of strain BT553^T^ is MT893357.

## Description of *Sphingomonas aurea* sp. nov.

*Sphingomonas aurea* (au’re.a. L. fem. adj. *aurea,* golden).

Cells are Gram-stain-negative, non-motile and rod-shaped. Colonies grown on NA agar have a slightly convex, circular and glistening appearance with orange-yellow colouration after 72 h of incubation at 30 °C. The cells range in size from ~0.6–0.8 µm in width and 1.4–1.8 µm in length. The strain grows on NA agar at temperatures between 25–30 °C (optimum at 25 °C) and tolerates NaCl concentrations of 0–2.5 % (optimal at 0%). It exhibits growth on NA, TSA, LB and R2A agar but not MAC agar. Oxidase activity is positive, but catalase activity is negative. In API 20NE testing, positive reactions for *β*-glucosidase (aesculin hydrolysis) and *β*-galactosidase (PNPG). It tests negative for arginine dihydrolase, nitrate reduction, indole production and acid production from glucose, urease, protease (gelatin hydrolysis), d-glucose, l-arabinose, d-mannose, d-mannitol, *N*-acetyl-d-glucosamine, d-maltose, gluconate, caprate, adipate, l-malate, citrate and phenylacetate. In API ZYM testing, it tests positive for alkaline phosphatase, leucine arylamidase, *α*-chymotrypsin, acid phosphatase, naphtol-AS-BI-phosphohydrolase, *α*-galactosidase, *β*-galactosidase (ONPG) and *N*-acetyl-*β*-glucosaminidase, with weakly positive reactions for esterase (C4), esterase (C8), valine arylamidase, cystine arylamidase, trypsin, *α*-glucosidase (starch hydrolysis) and *β*-glucosidase. It tests negative for lipase (C14), *β*-glucuronidase, *α*-mannosidase and *α*-fucosidase.

The major respiratory quinone is Q-10. Dominant cellular fatty acids include summed feature 3 (C_16:1_ ω7c/C_16:1_ ω6c) and summed feature 8 (C_18:1_ ω7c/C_18:1_ ω6c). The major polyamine is homospermidine (85.34%), with putrescine (14.66%) as the minor polyamine. The major polar lipids in this strain include DPG, PG, PE, PC, SL and glycosphingolipid.

The type strain for *Sphingomonas aurea*, KR1UV-12^T^ (=KCTC 92959^T^ = TBRC 18506^T^), was isolated from soil in the Republic of Korea. The genome sequence of strain KR1UV-12^T^ has been deposited in GenBank/DDBJ/EMBL under the accession number JAUUDS000000000. The GenBank accession number for the 16S rRNA gene sequence of strain KR1UV-12^T^ is OR342729.

## Supplementary material

10.1099/ijsem.0.006572 Uncited Supplementary Material 1.

## Abbreviations

ANI: average nucleotide identity
blast: basic local alignment search tool
CDSs: coding sequences
dDDH: digital DNA–DNA hybridization
DPG: diphosphatidylglycerol
GTDB: Genome Taxonomy Database
LB: lysogeny broth
ML: maximum likelihood
MP: maximum parsimony
NA: nutrient agar
NCBI: National Center for Biotechnology Information
NJ: neighbour joining
PC: phosphatidylcholine
PE: phosphatidylethanolamine
PG: phosphatidylglycerol
PL: phospholipids
R2A: Reasoner’s 2A
RAST: Rapid Annotation using Subsystem Technology
SL: sphingolipid
TSA: tryptic soy agar
UBCG: up-to-date bacterial core gene
