# Supplementary material for: Sphingomonas longa sp. nov., Sphingomonas mollis sp. nov. and Sphingomonas aurea sp. nov.: three novel Sphingomonas species isolated from soil
Source: Int J Syst Evol Microbiol. 2024 Dec 18;74(12):006572. doi: 10.1099/ijsem.0.006572 (PMC12453569; doi:10.1099/ijsem.0.006572)

Table S1. *Sphingomonas* strains included in this study

Culture collections are abbreviated as KCTC, Korean Collection for Type Cultures, Taejon, Korea; KACC, Korean Agricultural Culture Collection, Wanju, Korea; NBRC, Biological Resource Center, NITE, Shibuya, Japan; TBRC, Thailand Bioresource Research Center, Pathum Thani, Thailand; DSM, Deutsche Sammlung von Mikroorganismen, Braunschweig, German; JCM, Japan Collection of Microorganisms, Tsukuba, Japan.

**Strain**	**Other designation**	**Source**	**G+C content (mol%)**
BT552 (= KCTC 82094^{T})	NBRC 114993^{T}	Soil, Korea	66.8
BT553 (= KCTC 82095^{T})	NBRC 114994^{T}	Soil, Korea	65.9
KR1UV-12 (= KCTC 92959^{T})	TBRC 18506^{T}	Soil, Korea	68.3
Sphingomonas melonis DAPP-PG 224^{T}	DSM 14444^{T}	Fruits of yellow spanish melons, Spain	67.0
Sphingomonas aquatilis JSS7^{T}	KCTC 2883^{T}	Natural mineral water, Korea	67.1
Sphingomonas rubra BH3^{T}	JCM 16230^{T}	Bioreactor wastewater, China	68.8
Sphingomonas liriopis RP10^{T}	TBRC 15161^{T}	Liriope platyphylla fruit, Korea	68.2
Sphingomonas metalli 9O-5^{T}	KCTC 42759^{T}	Abandoned lead–zinc mine, China	68.6
Sphingomonas cynarae SPC-1^{T}	JCM 17498^{T}	Phyllosphere of wild cardoon, Italy	66.6
Sphingomonas kyungheensis THGp-B283^{T}	KACC 16224^{T}	Soil of a ginseng field, Korea	68.3

Table S2. Genome assembly statistics of strain BT552^{T}, BT553^{T} and the closely related *Sphingomonas* species

Data on the related type strains were retrieved from GenBank (https://www.ncbi.nlm.nih.gov/genbank/).

Taxa: 1, strain BT552^{T}; 2, *S. melonis* DAPP-PG 224^{T}; 3, *S. aquatilis* JSS7^{T}; 4, *S. kyungheensis* THG-B283^{T}; 5, *S. liriopis* RP10^{T}; 6, *S.cynarae* SPC-1^{T};

	BT552	BT553	melonis	aquatilis	kyungheensis	liriopis	taxi	cynarae
Genome size (bp)	4,035,561	3,941,714	3,944,577	3,591,671	4,055,944	3,555,040	3,859,099	3,941,891
Total genes number	3,877	3,751	3,298	3,457	3,802	3,441	3,804	5,449
G+C content (%)	66.8	65.9	67.0	67.1	68.3	68.2	67.9	66.6
N50	707,936	374,641	3,820,529	447,938	673,089	167,178	3,859,099	544,746
Number of contigs	10	24	6	36	28	37	3	27
Protein coding genes (CDS)	3,804	3,648	3,236	3,383	3,731	3,342	3,714	3,821
RNA genes	50	48	45	50	54	54	64	51
Completeness (%)	100	98.28	100	100	100	100	100	100
Contamination (%)	0	0	1.72	0	0	0	0	0

Table S3. Genome assembly statistics of strain KR1UV-12^T and the closely related *Sphingomonas* species

Data on the related type strains were retrieved from GenBank (https://www.ncbi.nlm.nih.gov/genbank/).

Taxa: 1, strain KR1UV-12^T; 2, *S. melonis* DAPP-PG 224^T; 3, *S. rubra* CGMCC 1.9113^T; 4, *S. liriopis* RP10^T; 5, *S. aquatilis* JSS7^T; 6. *S.metalli* 9O-5^T;

	1	2	3	4	5	6
Genome size (bp)	3,418,792	3,944,577	3,200,202	3,555,040	3,591,671	3,855,782
Total genes number	3,298	3,298	3,194	3,441	3,457	5,449
G+C content (%)	68.3	67	68.8	68.2	67.1	68.6
N50	317,083	3,820,529	252,988	167,178	447,938	894,380
Number of contigs	15	6	25	37	36	21
Protein coding genes (CDS)	3,236	3,236	3,092	3,342	3,383	3,706
RNA genes	45	45	71	54	50	54
Completeness (%)	100	100	100	100	100	99.14
Contamination (%)	0	1.72	0	0	0	0

Table S4. The average nucleotide identity (ANI) analysis and digital DNA-DNA hybridization analysis of the whole genome between strain BT552^{T} and related type strains of *Sphingomonas* species for which the genome has been published. Data of related type strains were taken from NCBI (https://www.ncbi.nlm.nih.gov/)

			**BT552**	
**Species**	**Strain**	**Genome**	**ANI (%)**	**DDH(%)**
Sphingomonas rubra	CGMCC 1.9113^{T}	GCA 900115745	77.64	22.00
Sphingomonas cynarae	JCM 17498^{T}	GCA 039537815	77.36	22.10
Sphingomonas abaci	DSM 15867^{T}	GCA 014199625	77.07	21.70
Sphingomonas metalli	CGMCC 1.15330^{T}	GCA 014641735	76.96	21.60
Sphingomonas liriopis	RP10^{T}	GCA 024211255	76.64	21.70
Sphingomonas taxi	ATCC 55669^{T}	GCA 000764535	76.35	21.20
Sphingomonas insulae	KCTC 12872^{T}	GCA 010450875	76.13	21.40
Sphingomonas ginsenosidivorax	KHI67^{T}	GCA 007995065	75.99	20.90
Sphingomonas kyungheensis	THG-B283^{T}	GCA 037120395	75.98	21.20
Sphingomonas jinjuensis	YC6723^{T}	GCA 014197105	75.97	21.00
Sphingomonas carotinifaciens	DSM 27347^{T}	GCA 009789535	75.88	20.70
Sphingomonas aerolata	NW12^{T}	GCA 003046295	75.63	20.20
Sphingomonas aquatilis	DSM 15581^{T}	GCA 014196115	75.57	20.90
Sphingomonas melonis	DAPP-PG 224^{T}	GCA 000379045	75.46	20.80
Sphingomonas pseudosanguinis	DSM 19512^{T}	GCA 014196255	75.35	20.80
Sphingomonas citri	RRHST34^{T}	GCA 019429485	75.19	20.80
Sphingomonas parapaucimobilis	NBRC 15100^{T}	GCA 000787715	75.17	20.90
Sphingomonas yabuuchiae	DSM 14562^{T}	GCA 014199595	75.11	20.80
Sphingomonas sanguinis	NBRC 13937^{T}	GCA 001591005	74.98	20.50
Sphingomonas ginsenosidimutans	KACC 14949^{T}	GCA 002374835	74.95	20.40
Sphingomonas zeae	DSM 100049^{T}	GCA 014197135	74.89	20.40
Sphingomonas paucimobilis	NBRC 13935^{T}	GCA 000739895	74.86	20.70
Sphingomonas palmae	JS21-1^{T}	GCA 900109565	74.73	20.30
Sphingomonas gellani	S6-262^{T}	GCA 900110035	74.70	20.70
Sphingomonas hominis	HHU CXW^{T}	GCA 013328205	74.32	20.20
Sphingomonas lenta	1PNM-20^{T}	GCA 002288825	74.27	19.90
Sphingomonas mali	NBRC 15500^{T}	GCA 001598415	72.78	19.90

Table S5. The average nucleotide identity (ANI) analysis and digital DNA-DNA hybridization analysis of the whole genome between strain BT553^{T} and related type strains of *Sphingomonas* species for which the genome has been published. Data of related type strains were taken from NCBI (https://www.ncbi.nlm.nih.gov/)

			**BT553**	
**Species**	**Strain**	**Genome**	**ANI (%)**	**DDH(%)**
Sphingomonas cynarae	JCM 17498^{T}	GCA 039537815	79.90	23.70
Sphingomonas rubra	CGMCC 1.9113^{T}	GCA 900115745	77.65	21.80
Sphingomonas abaci	DSM 15867^{T}	GCA 014199625	76.91	21.40
Sphingomonas metalli	CGMCC 1.15330^{T}	GCA 014641735	76.84	21.50
Sphingomonas liriopis	RP10^{T}	GCA 024211255	76.39	21.30
Sphingomonas taxi	ATCC 55669^{T}	GCA 000764535	76.11	21.00
Sphingomonas ginsenosidivorax	KHI67^{T}	GCA 007995065	76.09	21.20
Sphingomonas carotinifaciens	DSM 27347^{T}	GCA 009789535	75.90	20.70
Sphingomonas insulae	KCTC 12872^{T}	GCA 010450875	75.85	21.10
Sphingomonas kyungheensis	THG-B283^{T}	GCA 037120395	75.84	20.90
Sphingomonas aquatilis	DSM 15581^{T}	GCA 014196115	75.71	20.80
Sphingomonas aerolata	NW12^{T}	GCA 003046295	75.66	20.60
Sphingomonas melonis	DAPP-PG 224^{T}	GCA 000379045	75.61	20.60
Sphingomonas pseudosanguinis	DSM 19512^{T}	GCA 014196255	75.45	20.40
Sphingomonas parapaucimobilis	NBRC 15100^{T}	GCA 000787715	75.33	20.40
Sphingomonas jinjuensis	YC6723^{T}	GCA 014197105	75.26	20.40
Sphingomonas yabuuchiae	DSM 14562^{T}	GCA 014199595	75.16	20.40
Sphingomonas sanguinis	NBRC 13937^{T}	GCA 001591005	75.04	20.20
Sphingomonas paucimobilis	NBRC 13935^{T}	GCA 000739895	74.95	20.30
Sphingomonas zeae	DSM 100049^{T}	GCA 014197135	74.94	20.30
Sphingomonas ginsenosidimutans	KACC 14949^{T}	GCA 002374835	74.78	19.90
Sphingomonas gellani	S6-262^{T}	GCA 900110035	74.49	20.30
Sphingomonas citri	RRHST34^{T}	GCA 019429485	74.30	20.50
Sphingomonas palmae	JS21-1^{T}	GCA 900109565	74.27	19.80
Sphingomonas hominis	HHU CXW^{T}	GCA 013328205	74.24	19.80
Sphingomonas lenta	1PNM-20^{T}	GCA 002288825	73.62	19.60
Sphingomonas pruni	NBRC 15498^{T}	GCA 001598455	72.87	19.40

Table S6. The average nucleotide identity (ANI) analysis and digital DNA-DNA hybridization analysis of the whole genome between strain KR1UV-12^{T} and related type strains of *Sphingomonas* species for which the genome has been published. Data of related type strains were taken from NCBI (https://www.ncbi.nlm.nih.gov/)

			**KR1UV-12**	
**Species**	**Strain**	**Genome**	**ANI (%)**	**DDH(%)**
Sphingomonas metalli	CGMCC 1.15330^{T}	GCA 014641735	80.15	24.30
Sphingomonas rubra	CGMCC 1.9113^{T}	GCA 900115745	78.67	22.60
Sphingomonas cynarae	JCM 17498^{T}	GCA 039537815	78.14	22.50
Sphingomonas abaci	DSM 15867^{T}	GCA 014199625	77.96	22.70
Sphingomonas liriopis	RP10^{T}	GCA 024211255	77.20	22.00
Sphingomonas taxi	ATCC 55669^{T}	GCA 000764535	76.71	21.40
Sphingomonas carotinifaciens	DSM 27347^{T}	GCA 009789535	76.71	21.60
Sphingomonas kyungheensis	THG-B283^{T}	GCA 037120395	76.61	21.60
Sphingomonas jinjuensis	YC6723^{T}	GCA 014197105	76.57	21.30
Sphingomonas aquatilis	DSM 15581^{T}	GCA 014196115	76.37	21.30
Sphingomonas melonis	DAPP-PG 224^{T}	GCA 000379045	76.30	21.30
Sphingomonas insulae	KCTC 12872^{T}	GCA 010450875	76.30	21.60
Sphingomonas ginsenosidivorax	KHI67^{T}	GCA 007995065	76.24	21.00
Sphingomonas pseudosanguinis	DSM 19512^{T}	GCA 014196255	76.16	21.10
Sphingomonas parapaucimobilis	NBRC 15100^{T}	GCA 000787715	75.93	21.10
Sphingomonas yabuuchiae	DSM 14562^{T}	GCA 014199595	75.88	21.20
Sphingomonas sanguinis	NBRC 13937^{T}	GCA 001591005	75.78	20.90
Sphingomonas citri	RRHST34^{T}	GCA 019429485	75.74	21.30
Sphingomonas zeae	DSM 100049^{T}	GCA 014197135	75.59	20.90
Sphingomonas ginsenosidimutans	KACC 14949^{T}	GCA 002374835	75.58	20.90
Sphingomonas paucimobilis	NBRC 13935^{T}	GCA 000739895	75.54	21.00
Sphingomonas aerolata	NW12^{T}	GCA 003046295	75.47	20.60
Sphingomonas palmae	JS21-1^{T}	GCA 900109565	75.18	21.00
Sphingomonas gellani	S6-262^{T}	GCA 900110035	75.13	20.80
Sphingomonas hominis	HHU CXW^{T}	GCA 013328205	75.02	20.80
Sphingomonas lenta	1PNM-20^{T}	GCA 002288825	75.00	21.00
Sphingomonas pruni	NBRC 15498^{T}	GCA 001598455	73.22	20.10

Table S7. The amino acid similarity of auxin biosynthesis genes in strain BT552^{T} compared with related *Sphingomonas* species, strain BT553^{T}, and strain KR1UV-12^{T}

Taxa: 1, strain BT553^{T}; 2, strain KR1UV-12^{T}; 3, *S. melonis* DAPP-PG 224^{T}; 4, *S. aquatilis* JSS7^{T}; 5, *S. liriopis* RP10^{T}; 6, *S. kyungheensis* THG-B283^{T}; 7, *S.cynarae* SPC-1^{T};

No.	Auxin biosynthesis in strain BT552^{T}	Auxin biosynthesis similarity (%) with using BLASTp						
		1	2	3	4	5	6	7
1	Tryptophan synthase alpha chain (EC 4.2.1.20)	83.97	79.48	83.65	82.89	82.71	77.74	84.59
2	Tryptophan synthase beta chain (EC 4.2.1.20)	92.20	93.10	90.98	90.49	91.15	89.64	91.44
3	Anthranilate phosphoribosyltransferase (EC 2.4.2.18)	83.69	77.04	77.58	77.88	76.67	78.18	79.69
5	Phosphoribosylanthranilate isomerase (EC 5.3.1.24)	77.03	78.54	79.02	79.02	76.70	71.36	74.40

Table S8. The amino acid similarity of auxin biosynthesis genes in strain BT553^{T} compared with related *Sphingomonas* species, strain BT552^{T}, and strain KR1UV-12^{T}

Taxa: 1, strain BT552^{T}; 2, strain KR1UV-12^{T}; 3, *S. melonis* DAPP-PG 224^{T}; 4, *S. aquatilis* JSS7^{T}; 5, *S. liriopis* RP10^{T}; 6, *S. taxi* ATCC 55669^{T}; 7, *S.cynarae* SPC-1^{T};

No.	Auxin biosynthesis in strain BT553^{T}	Auxin biosynthesis similarity (%) with using BLASTp						
		1	2	3	4	5	6	7
1	Tryptophan synthase alpha chain (EC 4.2.1.20)	83.97	82.76	82.13	81.75	83.91	80.53	86.99
2	Tryptophan synthase beta chain (EC 4.2.1.20)	92.20	93.19	91.71	91.22	91.08	91.71	93.61
3	Anthranilate phosphoribosyltransferase (EC 2.4.2.18)	83.69	80.36	80.00	80.00	79.39	78.48	85.54
5	Phosphoribosylanthranilate isomerase (EC 5.3.1.24)	77.03	72.91	75.48	75.96	75.85	74.88	73.79

Table S9. The amino acid similarity of auxin biosynthesis genes in strain KR1UV-12^{T} compared with related *Sphingomonas* species, strain BT552^{T}, and strain BT553^{T}

Taxa: 1, strain BT552^{T}; 2, strain BT553^{T}; 3, *S. melonis* DAPP-PG 224^{T}; 4, *S. aquatilis* JSS7^{T}; 5, *S. liriopis* RP10^{T}; 6, *S. rubra* CGMCC 1.9113^{T}; 7, *S.metalli* 9O-1^{T};

No.	Auxin biosynthesis in strain KR1UV-12^{T}	Auxin biosynthesis similarity (%) with using BLASTp						
		1	2	3	4	5	6	7
1	Tryptophan synthase alpha chain (EC 4.2.1.20)	79.48	82.76	77.10	77.10	80.46	79.55	86.36
2	Tryptophan synthase beta chain (EC 4.2.1.20)	93.10	93.19	90.02	90.02	91.92	90.26	93.11
3	Anthranilate phosphoribosyltransferase (EC 2.4.2.18)	77.04	80.36	78.79	78.48	78.42	78.48	80.66
5	Phosphoribosylanthranilate isomerase (EC 5.3.1.24)	78.54	72.91	80.77	81.73	80.68	78.95	83.10

Table S10. The amino acid similarity of ammonia assimilation-related genes in strain BT552^T compared with related *Sphingomonas* species, strain BT553^T, and strain KR1UV-12^T

Taxa: 1, strain BT553^T; 2, strain KR1UV-12^T; 3, *S. melonis* DAPP-PG 224^T; 4, *S. aquatilis* JSS7^T; 5, *S. liriopis* RP10^T; 6, *S. kyungheensis* THG-B283^T; 7, *S.cynarae* SPC-1^T;

No.	Ammonia assimilation in strain BT552^T	Ammonia assimilation similarity (%) with using BLASTp						
		1	2	3	4	5	6	7
1	[Protein-PII] uridylyltransferase (EC 2.7.7.59)	77.79	77.68	77.24	76.15	81.81	82.03	81.22
2	Ammonium transporter	55.63	55.63	55.48	55.02	77.57	91.89	75.74
3	Asparagine synthetase [glutamine-hydrolyzing] (EC 6.3.5.4) AsnH	76.43	76.59	76.91	76.75	0.00	0.00	0.00
4	Aspartate aminotransferase (EC 2.6.1.1)	28.65	28.65	28.83	28.91	28.80	87.72	29.85
5	Aspartate racemase (EC 5.1.1.13)	0.00	0.00	0.00	0.00	61.23	0.00	64.16
6	Glutamate racemase (EC 5.1.1.3)	78.71	79.09	76.05	75.48	71.10	86.04	83.02
7	Glutamate synthase [NADPH] large chain (EC 1.4.1.13)	88.76	88.76	88.36	88.23	92.82	93.81	92.66
8	Glutamate synthase [NADPH] small chain (EC 1.4.1.13)	84.17	84.17	84.38	84.58	87.92	88.51	87.47
9	Glutamate-ammonia-ligase adenylyltransferase (EC 2.7.7.42)	71.65	71.54	73.14	72.16	72.77	76.24	77.00
10	Glutaminase (EC 3.5.1.2)	0.00	0.00	0.00	0.00	0.00	51.64	0.00
11	Glutamine synthetase type I (EC 6.3.1.2)	91.63	91.63	89.57	90.62	93.46	94.89	95.11
12	NAD-specific glutamate dehydrogenase (EC 1.4.1.2), large form	77.15	77.02	77.71	78.56	79.21	81.05	80.00
13	Nitrogen regulatory protein P-II	97.32	97.32	96.43	96.43	97.32	95.54	97.32

Table S11. The amino acid similarity of ammonia assimilation-related genes in strain BT553^{T} compared with related *Sphingomonas* species, strain BT552^{T}, and strain KR1UV-12^{T}

Taxa: 1, strain BT552^{T}; 2, strain KR1UV-12^{T}; 3, *S. melonis* DAPP-PG 224^{T}; 4, *S. aquatilis* JSS7^{T}; 5, *S. liriopis* RP10^{T}; 6, *S. taxi* ATCC 55669^{T}; 7, *S.cynarae* SPC-1^{T};

No.	Ammonia assimilation in strain BT553^{T}	Ammonia assimilation similarity (%) with using BLASTp						
		1	2	3	4	5	6	7
1	[Protein-PII] uridylyltransferase (EC 2.7.7.59)	79.41	79.52	79.06	78.92	88.81	82.03	84.77
2	Ammonium transporter	54.71	54.71	54.79	55.28	74.38	89.87	76.55
3	Asparagine synthetase [glutamine-hydrolyzing] (EC 6.3.5.4)	0.00	0.00	0.00	39.57	87.56	0.00	40.72
4	Aspartate aminotransferase (EC 2.6.1.1)	28.02	28.02	28.53	84.71	28.22	87.72	28.28
5	Glutamate racemase (EC 5.1.1.3)	76.14	75.38	75.76	75.56	72.83	86.04	83.77
6	Glutamate synthase [NADPH] large chain (EC 1.4.1.13)	88.19	88.13	88.81	88.61	93.61	93.81	91.92
7	Glutamate synthase [NADPH] small chain (EC 1.4.1.13)	83.58	83.78	83.99	83.30	92.52	88.51	91.51
8	Glutamate-ammonia-ligase adenylyltransferase (EC 2.7.7.42)	71.06	71.17	71.80	72.57	80.86	76.24	78.82
9	Glutaminase (EC 3.5.1.2)	0.00	0.00	0.00	61.69	66.34	51.64	0.00
10	Glutamine synthetase type I (EC 6.3.1.2)	90.79	90.79	89.15	90.64	97.47	94.89	94.47
11	Leucine-responsive regulatory protein, regulator for leucine (or lrp) regulon and high-affinity branched-chain amino acid transport system	0.00	0.00	93.33	0.00	0.00	0.00	0.00
12	NAD-specific glutamate dehydrogenase (EC 1.4.1.2), large form	75.44	75.31	75.03	75.64	83.76	81.05	80.20
13	Nitrogen regulatory protein P-II	97.32	97.32	99.11	98.21	98.21	95.54	95.54

Table S12. The amino acid similarity of ammonia assimilation-related genes in strain KR1UV-12^{T} compared with related *Sphingomonas* species, strain BT552^{T}, and strain BT553^{T}

Taxa: 1, strain BT552^{T}; 2, strain BT553^{T}; 3, *S. melonis* DAPP-PG 224^{T}; 4, *S. aquatilis* JSS7^{T}; 5, *S. liriopis* RP10^{T}; 6, *S. rubra* CGMCC 1.9113^{T}; 7, *S.metalli* 9O-1^{T};

No.	Ammonia assimilation in strain KR1UV-12^{T}	Ammonia assimilation similarity (%) with using BLASTp						
		1	2	3	4	5	6	7
1	[Protein-PII] uridylyltransferase (EC 2.7.7.59)	78.73	78.84	78.99	77.85	88.77	81.22	84.77
2	Ammonium transporter	55.02	55.02	55.73	53.80	92.31	75.74	78.03
3	Asparagine synthetase [glutamine-hydrolyzing] (EC 6.3.5.4)	0.00	0.00	37.58	0.00	0.00	0.00	40.72
4	Aspartate aminotransferase (EC 2.6.1.1)	93.75	93.75	28.07	94.78	29.57	29.85	28.28
5	Aspartate racemase (EC 5.1.1.13)	0.00	0.00	0.00	0.00	64.09	64.95	0.00
6	Glutamate racemase (EC 5.1.1.3)	80.23	79.47	70.57	74.90	81.51	83.02	83.77
7	Glutamate synthase [NADPH] large chain (EC 1.4.1.13)	89.18	89.18	90.77	89.14	94.03	92.66	91.92
8	Glutamate synthase [NADPH] small chain (EC 1.4.1.13)	83.71	83.92	87.63	85.15	92.16	87.55	91.77
9	Glutamate-ammonia-ligase adenylyltransferase (EC 2.7.7.42)	70.89	70.99	77.93	71.96	83.63	77.00	78.82
10	Glutamine synthetase type I (EC 6.3.1.2)	92.72	92.72	91.91	92.34	94.68	95.11	94.47
12	NAD-specific glutamate dehydrogenase (EC 1.4.1.2), large form	73.70	73.37	80.31	73.83	85.50	80.07	80.26
13	Nitrogen regulatory protein P-II	98.21	98.21	96.43	96.43	99.11	97.32	95.54

Fig S1. Maximum-likelihood phylogenetic tree based on 16S rRNA gene sequences showing the position of strains BT552^{T}, BT553^{T} and KR1UV-12^{T} and other relative species of the genus *Sphingomonas*. Numbers at nodes are bootstrap percentages (> 70%) based on the Maximum-likelihood algorithms. *Rhizorhabdus argentea* SP1^{T} was used as an outgroup. Bar, 0.01 substitutions per nucleotide position.

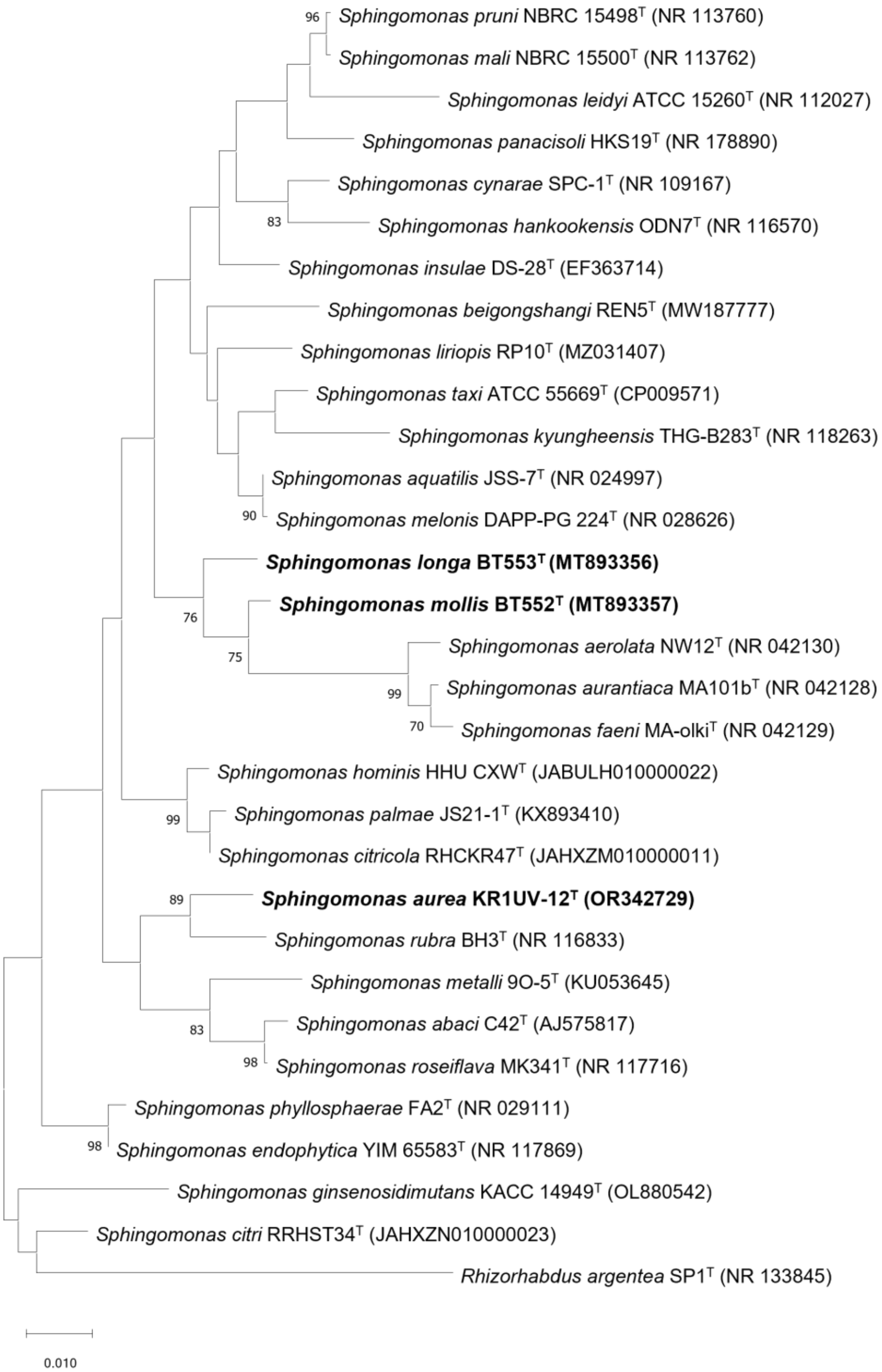

Fig S2. Maximum-parsimony phylogenetic tree based on 16S rRNA gene sequences showing the position of strains BT552^{T}, BT553^{T} and KR1UV-12^{T} and other relative species of the genus *Sphingomonas*. Numbers at nodes are bootstrap percentages (> 70%) based on the Maximum-parsimony algorithms. *Rhizorhabdus argentea* SP1^{T} was used as an outgroup. Bar, 0.01 substitutions per nucleotide position.

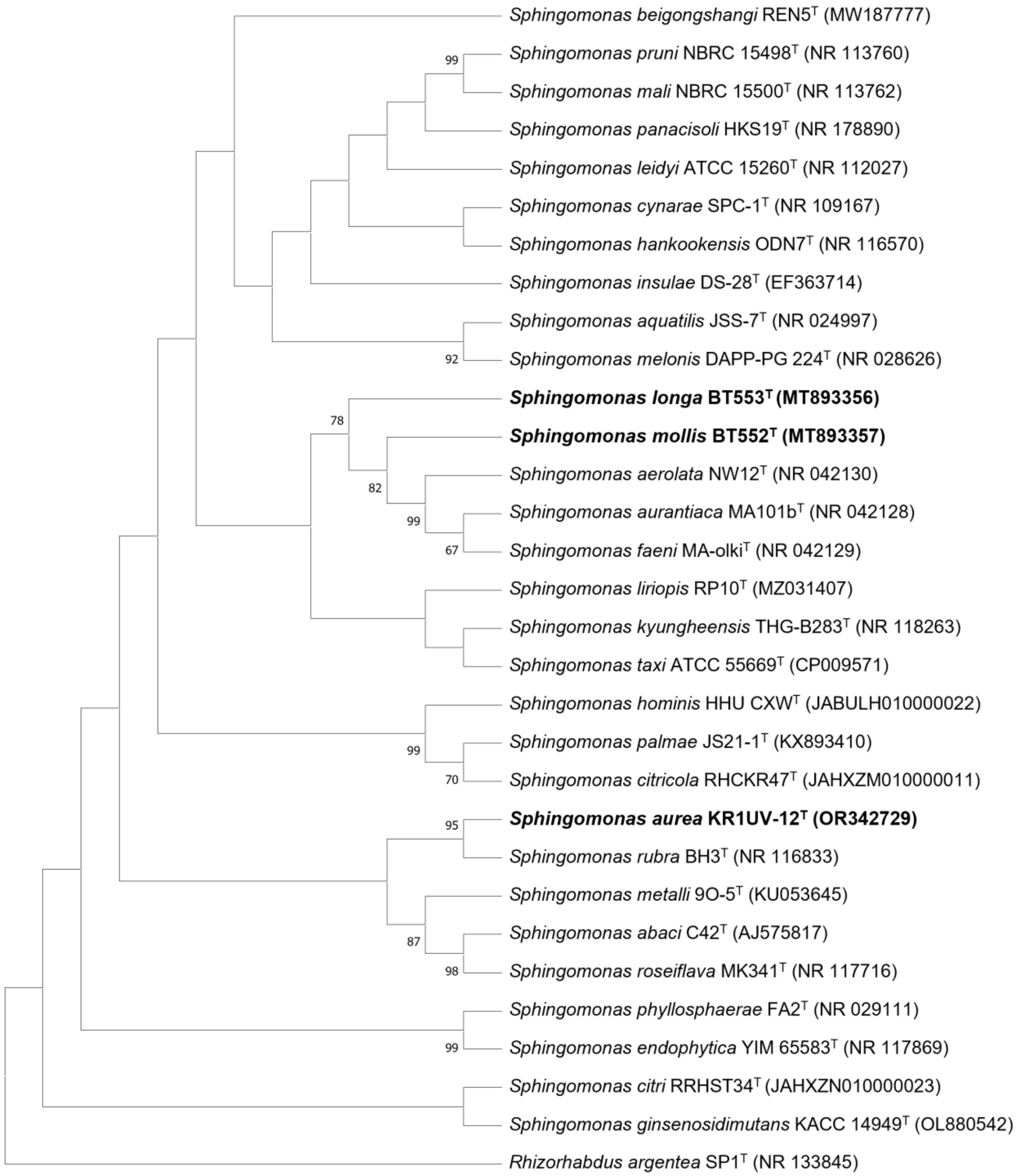

Fig S3. UBCG phylogenetic tree constructed from a comparative analysis of whole genome sequences showing the relationships of strain BT552^{T}, BT553^{T} and KR1UV-12^{T} with validly published species. *Blastomonas fulva* T2^{T} was used as an outgroup. Bar, 0.1 substitutions per position.

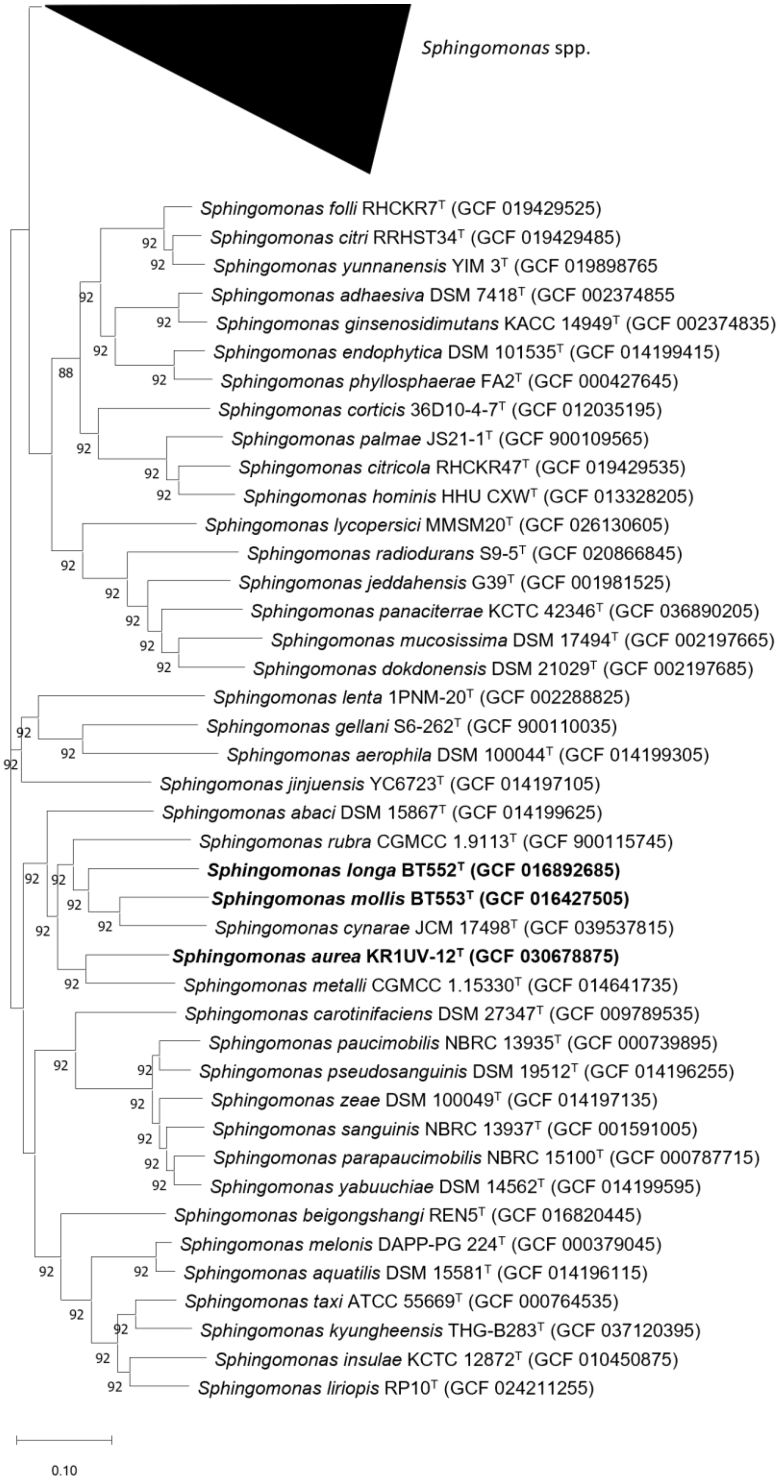

Fig S4. The subsystem category distribution of genes in strain BT552^{T}, expressed as percentages, detected by the RAST annotation server.

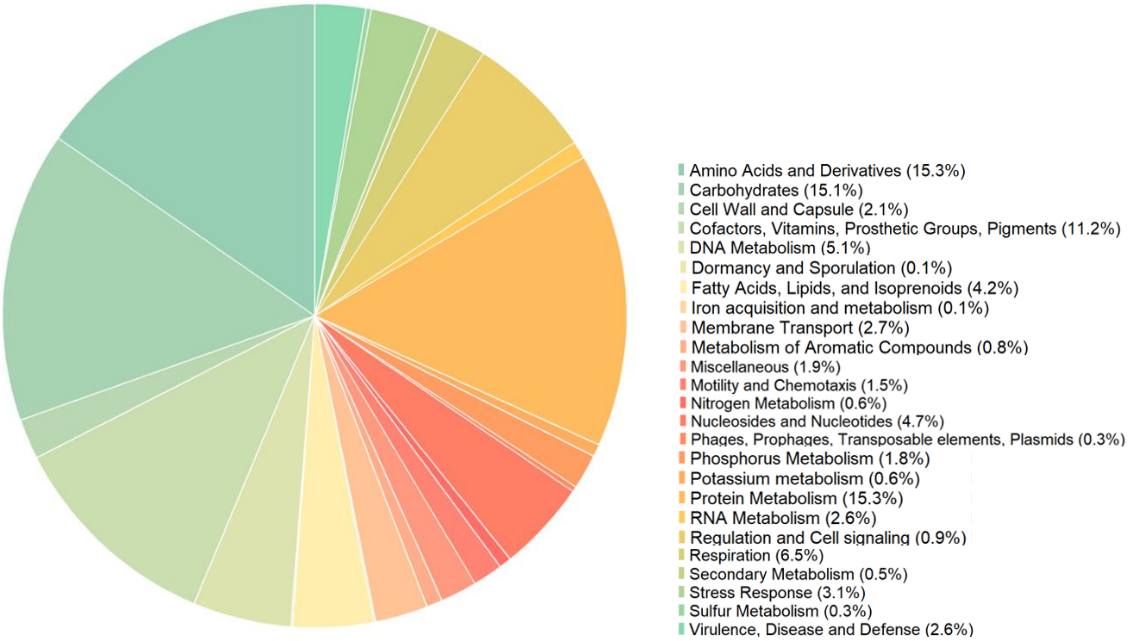

Fig S5. The subsystem category distribution of genes in strain BT553^T, expressed as percentages, detected by the RAST annotation server.

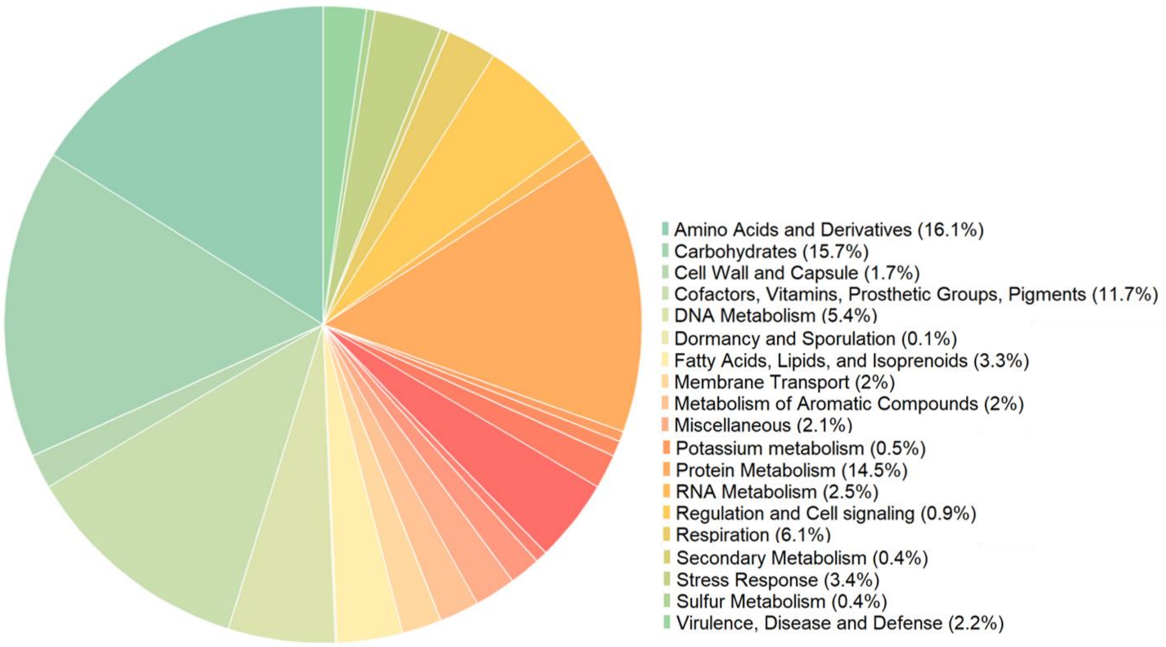

Fig S6. The subsystem category distribution of genes in strain KR1UV-12^{T}, expressed as percentages, detected by the RAST annotation server.

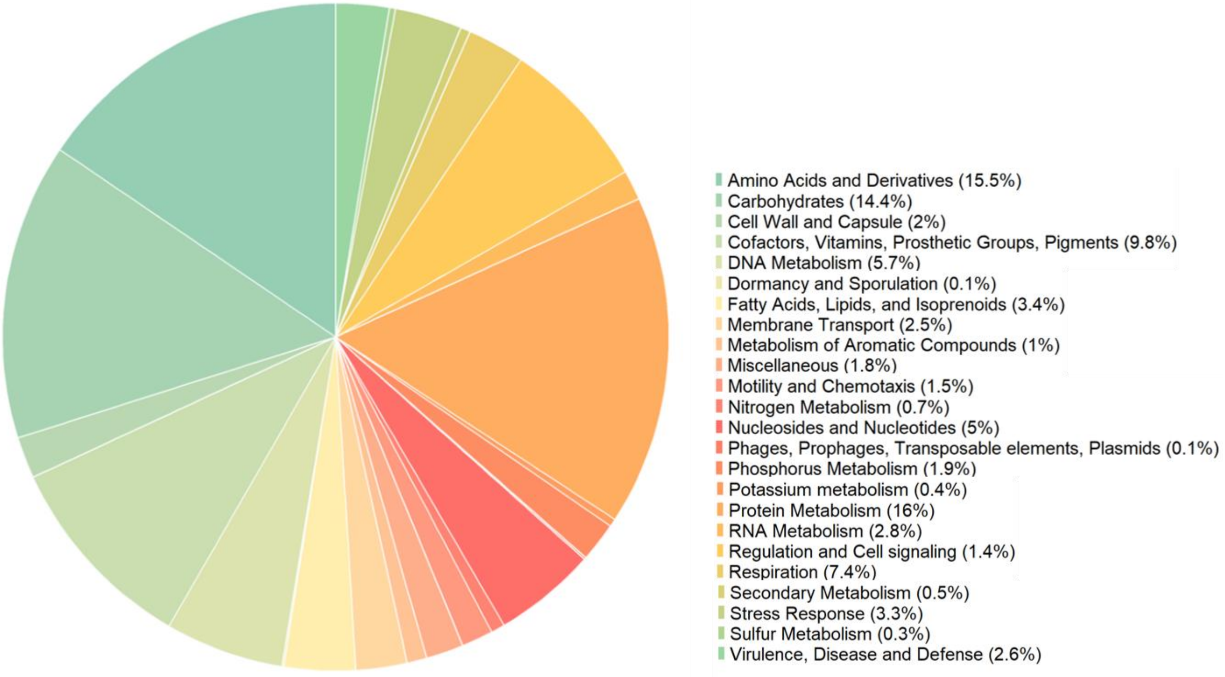

Fig S7. Polar lipid profile of strain BT552^T, after two-dimensional chromatography (a), total lipids; (b), phospholipids; (c), aminolipids; (d), glycolipids.

Abbreviations: DPG; diphosphatidylglycerol, PE; phosphatidylethanolamine, PG; phosphatidylglycerol, PC; phosphatidylcholine, PL; phospholipids, SGL; glycosphingolipid, AL; aminolipid, APL; aminophospholipid, L; lipids, SL; sphingolipid

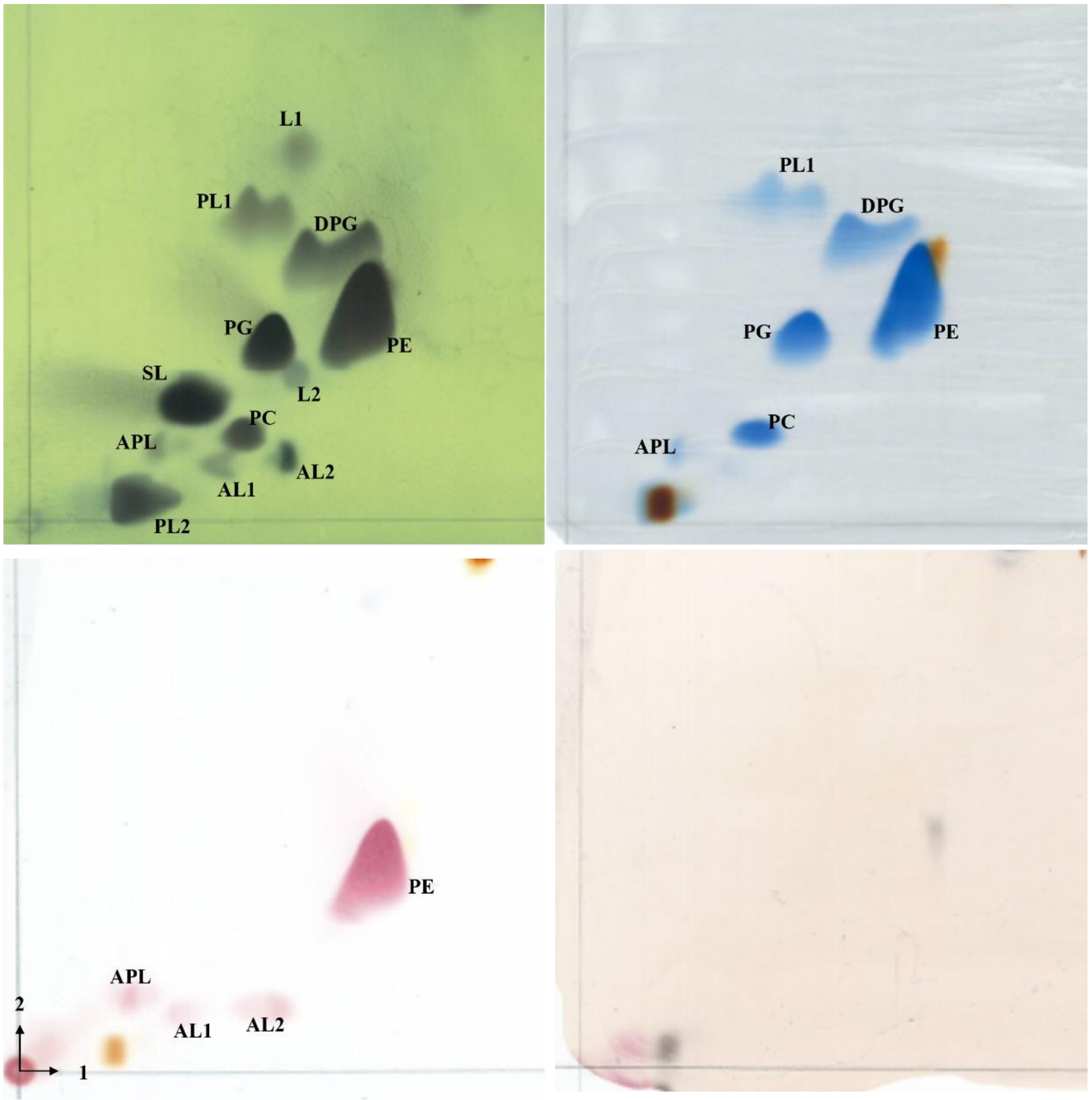

Fig S8. Polar lipid profile of strain BT553^T, after two-dimensional chromatography (a) total lipids; (b) phospholipids; (c) aminolipids; (d) glycolipids.

Abbreviations: DPG; diphosphatidylglycerol, PE; phosphatidylethanolamine, PG; phosphatidylglycerol, PC; phosphatidylcholine, PL; phospholipids, SGL; glycosphingolipid, AL; aminolipid, APL; aminophospholipid, L; lipids, SL; sphingolipid

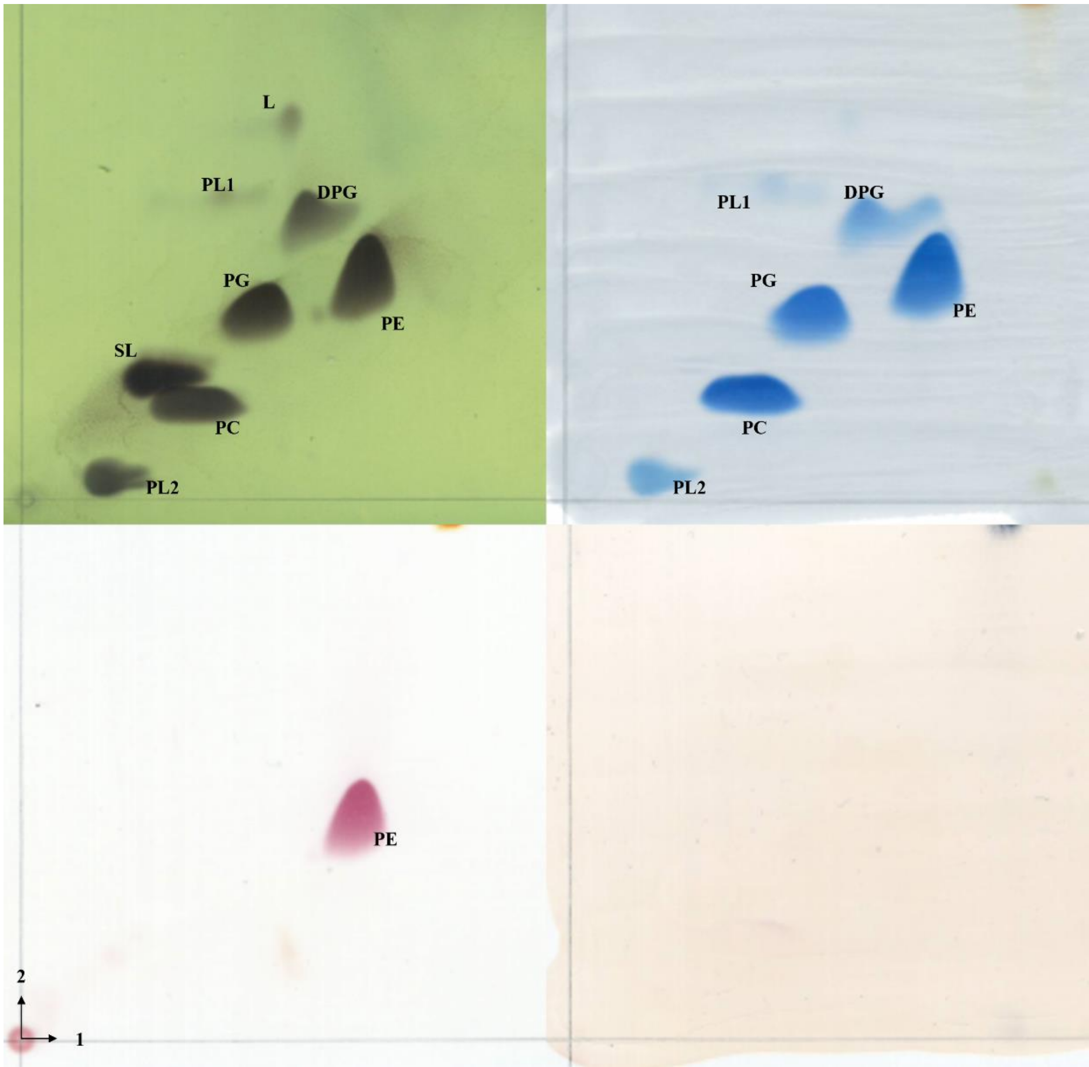

Fig S9. Polar lipid profile of strain KR1UV-12^{T}, after two-dimensional chromatography (a) total lipids; (b) phospholipids; (c) aminolipids; (d) glycolipids.

Abbreviations: DPG; diphosphatidylglycerol, PE; phosphatidylethanolamine, PG; phosphatidylglycerol, PC; phosphatidylcholine, PL; phospholipids, SGL; glycosphingolipid, AL; aminolipid, APL; aminophospholipid, L; lipids, SL; sphingolipid.

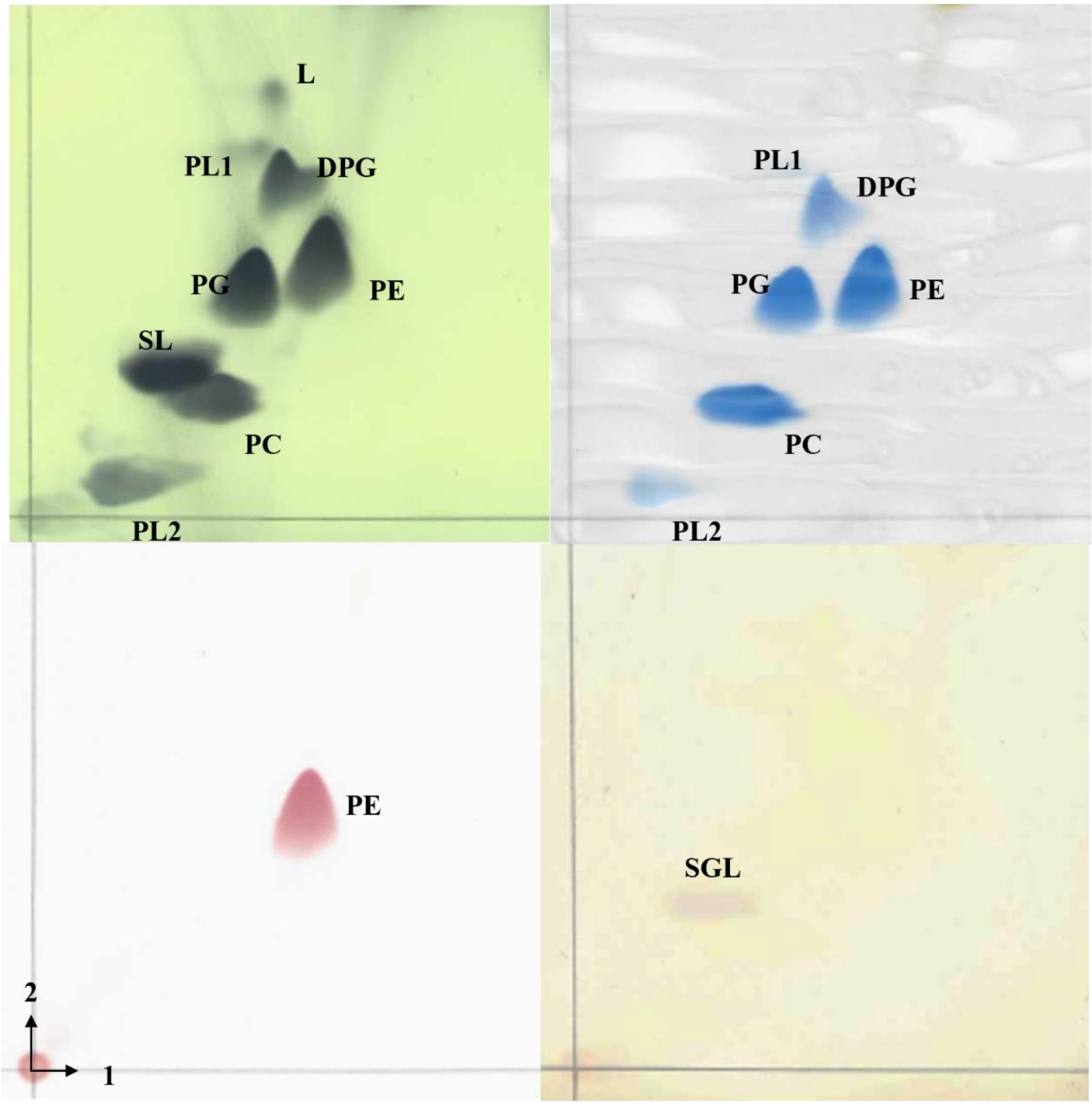

Fig S10. Analysis of Polyamine Profiles in strains BT552^T, BT553^T and KR1UV-12^T

(a) Polyamine standard retention times for strains BT552^T, BT553 ^{T}, KR1UV-12^T analysis. Peaks are identified as follows: Putrescine at RT 11.124 min, Spermidine at RT 15.481 min, Homospermidine at RT 18.269 min, and 2-hydroxy putrescine at RT 21.869 min; (b) Polyamine profile of strain BT552^T, with Putrescine detected at RT 11.095 min (6.48%) and Homospermidine at RT 18.151 min (93.52%); (c) Polyamine profile of strain BT553^T, with Spermidine detected at RT 15.945 min (1.93%) and Homospermidine at RT 18.866 min (98.07%); (d) Polyamine profile of strain KR1UV-12^T with Putrescine detected at RT 11.095 min (14.66%) and Homospermidine at RT 18.151 min (85.34%)

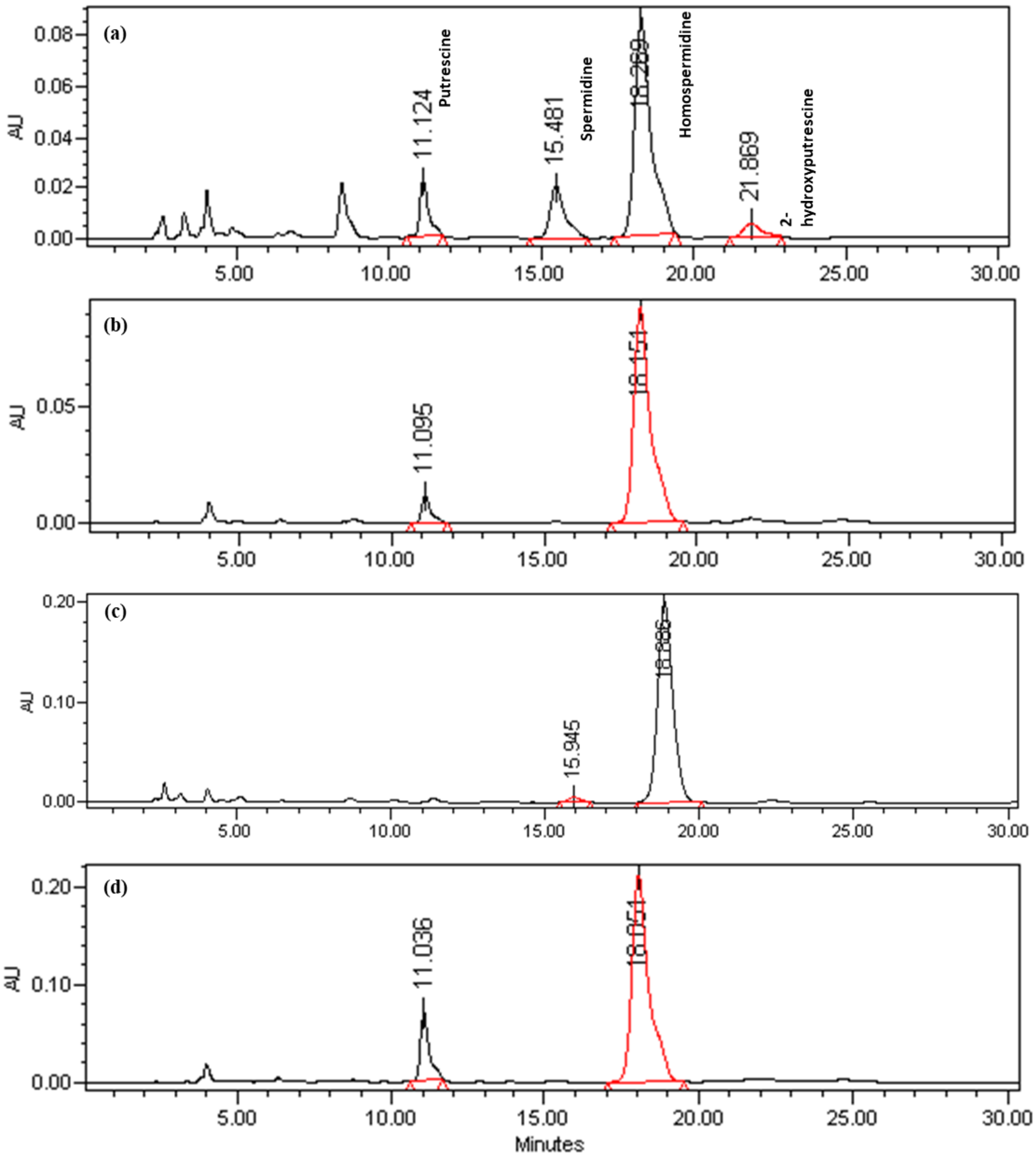

Supplement: Uncited Supplementary Material 1. [file ijsem-74-06572-s001.pdf]
